# Consensus‐Defined Sarcopenic Obesity and a Related Dynapenic Obesity Phenotype in Relation to Early Cognitive Impairment

**DOI:** 10.1002/jcsm.70395

**Published:** 2026-10-02

**Authors:** Mu‐Cyun Wang, Tao‐Chun Peng, Jeng‐Min Chiou, Yen‐Ching Chen, Jen‐Hau Chen

**Affiliations:** ^1^ Department of Geriatrics and Gerontology National Taiwan University Hospital Taipei Taiwan; ^2^ School of Medicine, College of Medicine National Taiwan University Taipei Taiwan; ^3^ Division of Geriatric Medicine, Department of Family and Community Medicine Tri‐Service General Hospital, National Defense Medical University Taipei Taiwan; ^4^ Institute of Statistics and Data Science National Taiwan University Taipei Taiwan; ^5^ Institute of Statistical Science Academia Sinica Taipei Taiwan; ^6^ Institute of Epidemiology and Preventive Medicine, College of Public Health National Taiwan University Taipei Taiwan; ^7^ Department of Public Health, College of Public Health National Taiwan University Taipei Taiwan; ^8^ Master Program of Sport Facility Management and Health Promotion National Taiwan University Taipei Taiwan; ^9^ Master Program of Statistics National Taiwan University Taipei Taiwan; ^10^ Department of Geriatrics and Gerontology National Taiwan University Hospital Yunlin Branch Yunlin Taiwan; ^11^ Department of Internal Medicine, College of Medicine National Taiwan University Taipei Taiwan

**Keywords:** cognitive impairment, dynapenic obesity, handgrip strength, muscle mass, obesity, sarcopenic obesity

## Abstract

**Background:**

Previous studies examining sarcopenic obesity in relation to cognitive function have utilized heterogeneous operational definitions. Some studies have evaluated low muscle strength combined with obesity, more appropriately described as dynapenic obesity, under the broader term sarcopenic obesity. We examined the associations of consensus‐defined sarcopenic obesity and a related strength‐based obesity phenotype with global and domain‐specific cognitive function in older adults and evaluated the robustness of these associations across alternative adiposity definitions.

**Methods:**

This prospective cohort study (2015–2019) included non‐demented older adults from the Taiwan Initiative for Geriatric Epidemiological Research. Participants underwent cognitive assessments at baseline and at the 2‐year follow‐up. Handgrip strength was measured using a dynamometer, and total muscle mass was measured by bioelectrical impedance analysis. Obesity was defined primarily as a body mass index ≥ 27 kg/m^2^. Sarcopenic obesity was defined as the coexistence of low muscle mass, low muscle strength and obesity, whereas dynapenic obesity was defined as low muscle strength and obesity. Multivariable‐adjusted generalized linear mixed models were used to examine the associations of sarcopenic obesity and a related dynapenic obesity phenotype with cognitive function across repeated measurements and over time.

**Results:**

At baseline, 366 older adults aged ≥ 69 years (54.6% women, mean ± standard deviation [SD] = 75.7 ± 4.8 years) were included. All 16 participants with dynapenic obesity also met the criteria for sarcopenic obesity. Sarcopenic obesity was associated with poor global cognition (MoCA‐T: $\hat{\beta }$ = −0.96; 95% confidence interval [CI] −1.80 to −0.13), memory (immediate free recall: $\hat{\beta }$ = −0.38; 95% CI, −0.72 to −0.04) and attention performance (Trail Making Test A: $\hat{\beta }$ = −0.43; 95% CI, −0.73 to −0.12). Dynapenic obesity was associated with poor global cognition (MoCA‐T: $\hat{\beta }$ = −1.15; 95% CI, −1.99 to −0.30), memory (immediate free recall: $\hat{\beta }$ = −0.43; 95% CI, −0.77 to −0.09) and attention performance (Trail Making Test A: $\hat{\beta }$ = −0.44; 95% CI, −0.75 to −0.14). The associations of sarcopenic obesity and dynapenic obesity with global cognition and attention performance remained evident among women and apolipoprotein E (*APOE*) ε4 non‐carriers.

**Conclusions:**

Sarcopenic obesity and a related dynapenic obesity phenotype were associated with poor global and domain‐specific cognitive function in non‐demented older adults. Muscle strength and obesity measures may facilitate early identification of these phenotypes and help identify individuals at increased risk of cognitive impairment. Future studies are needed to determine whether targeted interventions for sarcopenic and dynapenic obesity can prevent or delay cognitive decline.

## 1 Introduction

Sarcopenic obesity, defined as the coexistence of excess adiposity and reduced muscle mass and function [1], has gained attention as a distinct condition with adverse health implications (e.g., disability, functional decline, cardiometabolic diseases and mortality) [2]. While obesity is typically associated with increased cardiometabolic risk, its impact in older adults remains controversial, with some studies suggesting better survival, a phenomenon known as the “obesity paradox” [3]. However, such findings may mask important heterogeneity in body composition; in particular, the presence of sarcopenia in obese individuals likely affects health outcomes [1].

Age‐related changes in body composition, including reduced lean mass and increased fat mass, contribute to sarcopenic obesity through a bidirectional relationship: Obesity accelerates muscle loss via inflammation, insulin resistance and oxidative stress [4], whereas sarcopenia may promote fat accumulation by reducing energy expenditure [1]. Shared risk factors, including aging, inactivity and chronic disease, may further perpetuate this cycle.

Despite growing interest, prevalence estimates of sarcopenic obesity vary widely because of inconsistent definitions. A recent meta‐analysis reported a global prevalence of 11% in adults aged ≥ 60 years, which increased to 23% in those aged ≥ 75 years [5]. The lack of consensus until the recent definition released by the European Society for Clinical Nutrition and Metabolism (ESPEN) and the European Association for the Study of Obesity (EASO) in 2022 has limited comparability across earlier studies [6]. Moreover, dynapenic obesity, defined as the coexistence of excess adiposity and low muscle strength, has been used in the literature as a related but more readily assessed muscle–adiposity phenotype. However, it has often been used inconsistently or interchangeably with sarcopenic obesity, although sarcopenic obesity additionally requires evidence of reduced muscle mass under current consensus definitions [7].

Both sarcopenic obesity and cognitive impairment are common in aging populations. However, studies of sarcopenic obesity have used heterogeneous operational definitions. These include low muscle strength combined with high body mass index (BMI) or waist circumference [8, 9, 10], low muscle mass combined with high waist circumference or fat mass [10, 11, 12], and the coexistence of both low muscle mass and low muscle strength with high BMI or fat mass [13, 14]. Such variation in the muscle and adiposity components used to define sarcopenic obesity complicates comparisons across studies [15].

Although sarcopenia has been consistently associated with cognitive decline [16], findings concerning obesity and dementia have been mixed [17, 18] and may vary according to apolipoprotein E (*APOE*) ε4 status [19] and inflammatory profiles [20]. Studies of sarcopenic obesity have likewise yielded inconsistent results. Several studies have reported associations between different muscle–adiposity phenotypes and cognitive impairment [8, 9, 10, 11, 12, 13, 14], whereas two studies have reported no association between low muscle mass combined with high fat mass and cognitive impairment [21, 22]. Another study revealed that low muscle mass combined with low muscle strength and/or physical performance in the presence of a high fat mass index was not related to cognitive impairment, whereas low muscle strength combined with a high fat mass index was associated with poor global and domain‐specific cognition [23]. Moreover, relatively few cohort studies have applied standardized definitions to examine these relationships longitudinally [8, 14].

Because muscle strength is easier to assess than muscle mass in routine clinical and community settings, dynapenic obesity may represent a more readily assessed muscle–adiposity phenotype. However, muscle strength reflects muscle function rather than muscle quantity, and dynapenic obesity should therefore not be considered equivalent to sarcopenic obesity. Evidence remains limited regarding the associations of consensus‐defined sarcopenic obesity and related strength‐based obesity phenotypes with global and domain‐specific cognitive function in older adults.

The aim of this study was to examine the associations of sarcopenic obesity and the related, more readily assessed dynapenic obesity phenotype with global and domain‐specific cognitive function in older adults and to evaluate the robustness of these associations across alternative adiposity definitions.

## 2 Methods

### 2.1 Study Design and Participants

This prospective cohort study was based on the Taiwan Initiatives for Geriatric Epidemiological Research (TIGER), in which 605 community‐dwelling older adults were initially recruited from the senior health check‐up programme at National Taiwan University Hospital in 2011. In the study, 446 eligible participants were enrolled between 2015 and 2017. Of these participants, 80 were excluded because of prevalent dementia, neurological conditions, missing cognitive or sarcopenic‐obese data, or a BMI < 18.5 kg/m^2^, yielding a final sample of 366 for data analyses. We conducted follow‐up assessments 2 years later (2017–2019). The study flowchart is shown in Figure 1. This study was approved by the Research Ethics Committee of National Taiwan University Hospital (REC number: 201101039RB; 201112047RIB; 201312156RINC; 201412213RINC; 201712220RIN; 201712218RIN; 201812102RIN; 202012214RIN; 202012285RINA; 202112042RINA; 202312052RINC; 202401172RINC; 202501209RINB), and written informed consent was obtained from all participants. This study complies with the Helsinki Declaration of 1975.

**FIGURE 1:**
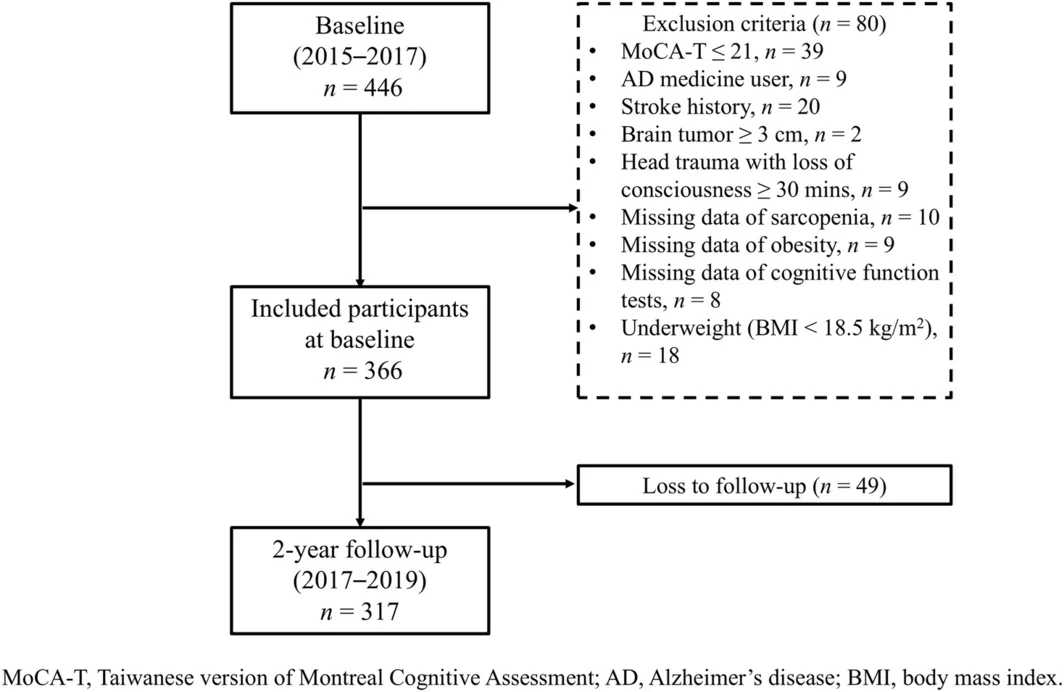
Flowchart of study population selection.

### 2.2 Sarcopenic Obesity and Dynapenic Obesity

Sarcopenic obesity is defined as the coexistence of low muscle mass, low muscle strength and obesity [6], whereas dynapenic obesity is defined as the coexistence of low muscle strength and obesity [24]. Total muscle mass (kg) was assessed using bioelectrical impedance analysis (BIA; Tanita BC420‐MA, Japan) and normalized by body weight (kg); participants in the lowest sex‐specific quartile were classified as having low muscle mass [25, 26]. Muscle strength was assessed using handgrip strength (Lafayette dynamometer, USA), with the Asian Working Group for Sarcopenia (AWGS) cutoffs (< 28 kg for older men and < 18 kg for older women) [27]. Obesity was defined primarily as a BMI ≥ 27 kg/m^2^ according to the adult obesity criterion established by the Taiwan Ministry of Health and Welfare [28]. This ethnicity‐ and population‐specific threshold was selected instead of the conventional BMI ≥ 30 kg/m^2^ criterion. Alternative definitions of obesity were based on central adiposity and fat mass. Central obesity was defined using Asian‐specific waist circumference cutoffs (≥ 90 cm for men and ≥ 80 cm for women) [29]. Fat mass index was calculated as body impedance analysis (BIA)‐assessed fat mass divided by height squared (kg/m^2^), with obesity defined as the highest sex‐specific quartile [30]. Because evidence supporting height‐adjusted fat mass measures remains limited, this definition was used only in sensitivity analyses [6].

### 2.3 Cognitive Assessments

Cognitive function was evaluated at baseline and follow‐up using a battery of neuropsychological tests administered by trained interviewers. Global cognition was assessed using the Montreal Cognitive Assessment‐Taiwanese version (MoCA‐T) [31]. Domain‐specific measures included immediate and delayed recall (Wechsler Logical Memory), attention (Trail Making Test A and digit span tests), executive function (Trail Making Test B) and verbal fluency (semantic categories). Domain scores were standardized into *z* scores. The Trail Making Test scores were reverse‐coded so that lower values indicated poorer performance.

### 2.4 Covariates

Sociodemographic data, lifestyle factors and comorbidities, including smoking, alcohol consumption, hypertension, diabetes, dyslipidemia, cardiovascular disease and depressive symptoms, were collected via structured questionnaires. Depressive symptoms were considered present if participants met any of the following criteria: a self‐reported depression history, use of antidepressant medication or a score ≥ 16 on the 20‐item Center for Epidemiologic Studies Depression (CES‐D) scale. Physical activity was measured using the International Physical Activity Questionnaire (IPAQ), expressed in metabolic equivalent of task (MET) minutes a week [32]. Functional status was assessed using the Barthel Index for basic activities of daily living (ADL) and the Lawton–Brody instrumental activities of daily living (IADL) scale. Diet quality was evaluated using the modified Alternative Healthy Eating Index [33].

### 2.5 Biomarkers

Serum inflammatory markers, such as C‐reactive protein (CRP) and interleukin‐6 (IL‐6), were measured in blood samples. Baseline CRP and IL‐6 concentrations were dichotomized at upper‐tertile cutoffs of > 1.2 mg/L and > 1.65 pg/mL, respectively. These thresholds were cohort‐specific and represented relatively high levels of low‐grade inflammation within this generally healthy, community‐dwelling population. The *APOE* ε4 genotype was determined on the basis of two single‐nucleotide polymorphisms, rs429358 and rs7412, using TaqMan SNP assays on an ABI 7900HT Fast Real‐Time PCR system (Applied Biosystems Inc., CA, USA). Individuals with at least one ε4 allele were categorized as *APOE* ε4 positive.

### 2.6 Statistical Analyses

Baseline characteristics were compared across sarcopenic obesity status and dynapenic obesity status using the Kruskal–Wallis test for continuous variables and the chi‐square test or Fisher's exact test for categorical variables. Generalized linear mixed models (GLMMs) with random intercepts were used to examine the associations of sarcopenic obesity and dynapenic obesity with cognitive outcomes over the 2‐year follow‐up period. Models were adjusted for potential confounders, including demographic characteristics, comorbidities, lifestyle factors, inflammatory markers and practice effects (defined as the number of completed cognitive assessments).

Two sets of models were evaluated. Model 1 included sarcopenic obesity status or dynapenic obesity status as a time‐varying exposure to examine associations between phenotype status and cognitive performance across repeated measurements. Model 2 included baseline phenotype status, follow‐up time and their interaction to assess whether cognitive performance over time differed according to baseline phenotype status.

Stratified analyses were conducted by sex, *APOE* ε4 status, diabetes status, and IL‐6 and CRP levels using multivariable‐adjusted GLMMs; these analyses were not adjusted for multiple comparisons. Several sensitivity analyses were performed. First, we repeated the analyses using alternative definitions of obesity, including central obesity defined as a waist circumference ≥ 90 cm for men and ≥ 80 cm for women and a high fat mass index defined as the highest sex‐specific quartile. Second, because the primary definition of low muscle mass was based on the lowest sex‐specific quartile of weight‐adjusted muscle mass, we conducted additional sensitivity analyses using alternative sex‐specific thresholds, including the lowest tertile and lowest quintile, to evaluate whether the findings were robust to the choice of internal cutoffs. Third, participants who had been excluded solely because of a BMI < 18.5 kg/m^2^ were re‐included, and the main GLMM analyses were repeated in this expanded analytic sample (*n* = 373).

Because muscle mass was standardized by body weight, the resulting measure was structurally related to BMI‐defined obesity. We explored whether the muscle mass component could be examined separately by presenting the distribution of these combinations descriptively rather than through a formal comparison between mass‐based and strength‐based phenotypes.

As previously mentioned, participants whose baseline cognitive data were missing or whose information was insufficient to determine sarcopenic obesity status were excluded. The mixed‐effects models included all available repeated observations; observations with missing outcome, exposure or covariate data at a given visit were excluded from the analysis for that visit. The number of missing values for each covariate is reported in Table 1.

**TABLE 1:** Characteristics of the study population by sarcopenic obesity status at baseline (2015–2017).

Variables	Missing *n*	Total (*n* = 366)	Nonsarcopenic‐nonobese (*n* = 303)	Sarcopenic‐nonobese (*n* = 20)	Nonsarcopenic‐obese (*n* = 27)	Sarcopenic‐obese (*n* = 16)	*p*
		Median (IQR)					
Age (year)	0	74.8 (71.8, 78.7)	74.7 (71.9, 78.0)	79.9 (73.7, 82.4)	72.9 (70.6, 76.2)	76.2 (71.4, 79.4)	**0.018**
Education (year)	0	14.0 (12.0, 16.0)	15.0 (12.0, 16.0)	14.0 (12.0, 16.0)	14.0 (11.0, 16.0)	12.0 (9.0, 16.0)	0.138
Physical activity (1000 MET‐min/week)	0	1.5 (0.8, 3.0)	1.6 (0.8, 3.1)	1.9 (1.0, 2.5)	2.8 (0.7, 4.0)	0.9 (0.1, 1.3)	**0.026**
ADL score	2	100 (100, 100)	100 (100, 100)	100 (100, 100)	100 (100, 100)	100 (100, 100)	0.688
IADL score	2	8 (8, 8)	8 (8, 8)	8 (8, 8)	8 (8, 8)	8 (8, 8)	0.757
mAHEI score	6	37.6 (31.7, 42.8)	37.8 (32.2, 42.9)	39.1 (31.4, 42.4)	36.4 (26.9, 43.8)	36.1 (25.5, 39.4)	0.432
Gait speed (m/s)	0	0.8 (0.6, 0.9)	0.8 (0.7, 0.9)	0.6 (0.5, 0.7)	0.8 (0.7, 0.9)	0.6 (0.6, 0.7)	**< 0.001**
Handgrip strength (kg)	0	22.0 (18.0, 30.0)	23.0 (18.0, 30.0)	14.3 (12.0, 22.0)	26.0 (20.0, 33.5)	16.0 (14.0, 16.8)	**< 0.001**
Weight‐adjusted total muscle mass (kg/kg)	0	0.7 (0.6, 0.7)	0.7 (0.6, 0.8)	0.6 (0.6, 0.7)	0.6 (0.5, 0.7)	0.6 (0.5, 0.6)	**< 0.001**
Fat mass (%)	0	27.4 (21.0, 33.1)	25.7 (20.0, 31.6)	37.2 (25.5, 38.2)	38.7 (26.7, 42.8)	39.9 (39.1, 43.8)	**< 0.001**
Fat mass index (kg/m^2^)	0	6.3 (4.8, 7.9)	5.8 (4.6, 7.1)	9.5 (6.5, 10.0)	10.6 (7.5, 12.7)	11.1 (10.7, 13.2)	**< 0.001**
BMI (kg/m^2^)	0	23.4 (21.6, 25.4)	22.9 (21.2, 24.3)	25.9 (25.4, 26.4)	28.3 (27.5, 31.0)	28.6 (27.7, 31.1)	**< 0.001**
Waist circumference (cm)	0	84.3 (78.0, 91.0)	83.0 (77.0, 88.5)	89.0 (85.3, 92.0)	95.0 (92.0, 99.0)	94.0 (90.0, 102.3)	**< 0.001**
C‐reactive protein (mg/L)	0	0.6 (0.3, 1.7)	0.5 (0.3, 1.4)	1.6 (0.6, 3.7)	1.5 (0.6, 3.2)	2.0 (1.2, 2.7)	**< 0.001**
Interleukin‐6 (pg/mL)	2	1.5 (1.2, 1.8)	1.5 (1.2, 1.8)	1.6 (1.4, 1.8)	1.4 (1.2, 1.7)	1.4 (1.3, 1.8)	0.519
		*n* (%)					
Women	0	200 (54.6)	158 (52.2)	13 (65.0)	16 (59.3)	13 (81.3)	0.092
Annual household income > 1 million (NTD)	9	75 (21.0)	60 (20.3)	7 (35.0)	6 (24.0)	2 (12.5)	0.355
Cigarette smoking	0	49 (13.4)	42 (13.9)	3 (15.0)	3 (11.1)	1 (6.3)	0.920
Alcohol consumption	0	48 (13.1)	38 (12.5)	4 (20.0)	4 (14.8)	2 (12.5)	0.704
*APOE* ε4 carriers	4	58 (16.0)	51 (16.9)	2 (11.1)	3 (11.1)	2 (12.5)	0.872
Depressive symptoms	0	25 (6.8)	18 (5.9)	3 (15.0)	1 (3.7)	3 (18.8)	0.072
Hypertension	0	230 (62.8)	186 (61.4)	15 (75.0)	18 (66.7)	11 (68.8)	0.583
Hyperlipidaemia	0	165 (45.1)	132 (43.6)	9 (45.0)	17 (63.0)	7 (43.8)	0.286
Diabetes mellitus	0	64 (17.5)	44 (14.5)	5 (25.0)	9 (33.3)	6 (37.5)	**0.007**
Heart disease	0	129 (35.3)	107 (35.3)	5 (25.0)	8 (29.6)	9 (56.3)	0.223

*Note: p* values were calculated by the Kruskal–Wallis test for continuous variables and the chi‐square test (or Fisher's exact test as appropriate) for categorical variables. The numbers in bold indicate significant findings (*p* < 0.05).

Abbreviations: ADL, activities of daily living; *APOE*, apolipoprotein E; BMI, body mass index; IADL, instrumental activities of daily living; IQR, interquartile range; mAHEI, modified Alternative Healthy Eating Index; MET, metabolic equivalent of task; NTD, new Taiwan dollar.

All the statistical tests were two‐sided, and a *p* value < 0.05 was considered to indicate statistical significance. Analyses were conducted using SAS version 9.4 (SAS Institute, Cary, NC, USA), and graphs were generated using R version 4.5.1.

## 3 Results

### 3.1 Characteristics of the Study Population

Among 366 older adults (median age 75 years, 55% women), 303 (83%) had neither sarcopenia nor obesity, 20 (5%) had sarcopenia alone, 27 (7%) had obesity alone and 16 (4%) had sarcopenic obesity (Table 1). Among the four groups (sarcopenic‐obese, nonsarcopenic‐obese, sarcopenic‐nonobese and nonsarcopenic‐nonobese), participants with sarcopenia alone were the oldest and exhibited the lowest handgrip strength and gait speed. Individuals with obesity alone had the greatest waist circumference. Participants with sarcopenic obesity had the lowest muscle mass, the highest BMI and the highest fat mass and fat mass index. The number of missing values for each covariate was small at baseline.

In terms of dynapenic obesity status, 204 participants (56%) had neither dynapenia nor obesity, 119 (33%) had dynapenia only, 27 (7%) had obesity only and 16 (4%) had dynapenic obesity (Table S1). Among the 303 participants classified as having neither sarcopenia nor obesity, 99 had dynapenia alone. All 16 obese participants with dynapenia also met the criteria for sarcopenia, resulting in complete overlap between the two phenotypes. Therefore, the corresponding estimates should be interpreted as findings for related, non‐independent muscle–adiposity phenotypes rather than as independent associations for two distinct groups. When obesity was alternatively defined using waist circumference, 23.8% of participants classified as having obesity alone under the BMI‐based definition were reclassified as having dynapenic obesity (data not shown).

### 3.2 Sarcopenic Obesity and Cognitive Function

In the multivariable GLMMs (Table 2), Model 1 (upper part) revealed that participants with sarcopenic obesity was associated with poorer global cognition (MoCA‐T: $\hat{\beta }$ = − 0.96; 95% CI: −1.80 to −0.13), memory (immediate free recall: $\hat{\beta }$ = − 0.38; 95% CI: −0.72 to −0.04) and attention performance (Trail Making Test A: $\hat{\beta }$ = − 0.43; 95% CI: −0.73 to −0.12) compared with those without sarcopenia or obesity. Obesity alone was associated with poorer executive function, as assessed by the Trail Making Test B ($\hat{\beta }$ = − 0.29; 95% CI: −0.56 to −0.01). In Model 2 (lower part), baseline sarcopenic obesity was associated with poorer global cognition, memory and attention performance, whereas baseline sarcopenia alone was associated with poorer attention. Baseline sarcopenic obesity showed a significant interaction with follow‐up time for the Trail Making Test B, suggesting that executive function over time was poorer in participants with baseline sarcopenic obesity. Baseline sarcopenia alone also showed a significant interaction with follow‐up time for the immediate theme and free recall, suggesting that memory performance over time was poorer in participants with baseline sarcopenia alone.

**TABLE 2:** Association between sarcopenic obesity status and cognitive function during 2015–2019.

Sarcopenic obesity status	Global cognitive function	Logical memory				Trail making test		Digit span		Verbal fluency
	MoCA‐T $\hat{\beta }$ (95% CI)	Immediate theme recall $\hat{\beta }$ (95% CI)	Immediate free recall $\hat{\beta }$ (95% CI)	Delayed theme recall $\hat{\beta }$ (95% CI)	Delayed free recall $\hat{\beta }$ (95% CI)	A $\hat{\beta }$ (95% CI)	B $\hat{\beta }$ (95% CI)	Forward $\hat{\beta }$ (95% CI)	Backward $\hat{\beta }$ (95% CI)	$\hat{\beta }$ (95% CI)
Nonsarcopenic‐nonobese	Reference	Reference	Reference	Reference	Reference	Reference	Reference	Reference	Reference	Reference
Sarcopenic‐nonobese	−0.34 (−1.04, 0.35)	0.30 (−0.02, 0.62)	−0.01 (−0.27, 0.24)	0.09 (−0.24, 0.42)	0.03 (−0.23, 0.30)	−0.09 (−0.32, 0.14)	−0.01 (−0.26, 0.25)	−0.16 (−0.42, 0.10)	−0.07 (−0.35, 0.21)	−0.01 (−0.26, 0.24)
Nonsarcopenic‐obese	−0.14 (−0.79, 0.50)	−0.09 (−0.40, 0.22)	−0.13 (−0.40, 0.15)	−0.18 (−0.50, 0.14)	−0.15 (−0.43, 0.13)	−0.18 (−0.43, 0.07)	**−0.29 (−0.56, −0.01)**	0.01 (−0.26, 0.27)	−0.02 (−0.29, 0.26)	−0.06 (−0.33, 0.21)
Sarcopenic‐obese	**−0.96 (−1.80, −0.13)**	−0.18 (−0.57, 0.22)	**−0.38 (−0.72, −0.04)**	−0.06 (−0.46, 0.35)	−0.21 (−0.56, 0.14)	**−0.43 (−0.73, −0.12)**	0.07 (−0.27, 0.41)	0.12 (−0.22, 0.45)	−0.16 (−0.51, 0.19)	−0.03 (−0.36, 0.30)
Nonsarcopenic‐nonobese	Reference	Reference	Reference	Reference	Reference	Reference	Reference	Reference	Reference	Reference
Sarcopenic‐nonobese	0.15 (−0.90, 1.20)	0.42 (−0.07, 0.91)	0.14 (−0.34, 0.61)	0.09 (−0.42, 0.60)	−0.07 (−0.55, 0.42)	0.02 (−0.39, 0.43)	**0.47 (0.001, 0.93)**	**−0.55 (−0.99, −0.11)**	−0.14 (−0.58, 0.31)	−0.11 (−0.59, 0.36)
Sarcopenic‐nonobese × time	0.27 (−0.39, 0.93)	**−0.33 (−0.61, −0.06)**	**−0.22 (−0.41, −0.02)**	−0.13 (−0.41, 0.15)	−0.11 (−0.31, 0.10)	0.09 (−0.09, 0.27)	0.03 (−0.17, 0.23)	0.05 (−0.17, 0.26)	0.07 (−0.17, 0.31)	−0.03 (−0.23, 0.16)
Nonsarcopenic‐obese	0.10 (−0.76, 0.97)	−0.004 (−0.41, 0.40)	−0.03 (−0.42, 0.36)	−0.22 (−0.64, 0.20)	−0.16 (−0.56, 0.24)	−0.10 (−0.44, 0.24)	−0.04 (−0.42, 0.35)	0.06 (−0.30, 0.43)	−0.003 (−0.37, 0.36)	0.06 (−0.33, 0.45)
Nonsarcopenic‐obese × time	0.20 (−0.32, 0.72)	0.14 (−0.08, 0.36)	−0.04 (−0.19, 0.12)	**0.23 (0.01, 0.45)**	0.07 (−0.09, 0.24)	0.01 (−0.13, 0.15)	−0.14 (−0.29, 0.02)	0.14 (−0.03, 0.31)	0.11 (−0.09, 0.30)	−0.02 (−0.17, 0.13)
Sarcopenic‐obese	**−1.13 (−2.24, −0.02)**	−0.37 (−0.89, 0.15)	**−0.53 (−1.03, −0.03)**	−0.23 (−0.77, 0.31)	−0.34 (−0.85, 0.18)	**−1.11 (−1.54, −0.67)**	−0.42 (−0.91, 0.07)	−0.03 (−0.49, 0.44)	−0.21 (−0.68, 0.26)	−0.10 (−0.61, 0.40)
Sarcopenic‐obese × time	−0.35 (−1.07, 0.36)	0.09 (−0.21, 0.39)	0.01 (−0.21, 0.23)	0.02 (−0.28, 0.33)	−0.07 (−0.30 0.15)	0.14 (−0.06, 0.34)	**−0.28 (−0.50, −0.06)**	0.14 (−0.10, 0.37)	−0.16 (−0.42, 0.10)	−0.04 (−0.25, 0.17)

*Note:* Generalized linear mixed models were used to estimate β coefficients for the cognitive variables (MoCA‐T scores or *z* scores of the cognitive domains). Adjusted for age, sex, education years, *APOE* ε4 status, depressive symptoms, follow‐up time, practice effect, cigarette smoking, alcohol consumption, hypertension, diabetes mellitus, dyslipidaemia, heart disease, physical activity by IPAQ, and interleukin‐6 and C‐reactive protein levels. The numbers in bold indicate significant findings (*p* < 0.05).

Abbreviations: CI, confidence interval; IPAQ, International Physical Activity Questionnaire; MoCA‐T, Montreal Cognitive Assessment‐Taiwanese version.

In exploratory stratified analyses, sarcopenic obesity was associated with poorer MoCA‐T performance among women, *APOE* ε4 non‐carriers and participants in the highest tertile of IL‐6 or CRP ($\hat{\beta }$ = − 1.93 to −0.89; all *p* < 0.05; Figure 2). It was also associated with poorer immediate free recall among men, *APOE* ε4 non‐carriers, participants without diabetes and participants in the highest tertile of CRP ($\hat{\beta }$ = − 0.82 to −0.36; all *p* < 0.05; Figure 3) and with poorer Trail Making Test A performance among women, *APOE* ε4 non‐carriers, participants without diabetes and participants in the lower two tertiles of IL‐6 ($\hat{\beta }$ = − 0.56 to −0.43; all *p* < 0.05; Figure 4). Obesity alone was associated with poorer Trail Making Test B performance in selected subgroups ($\hat{\beta }$ = − 1.14 to −0.32; all *p* < 0.05; data not shown).

**FIGURE 2:**
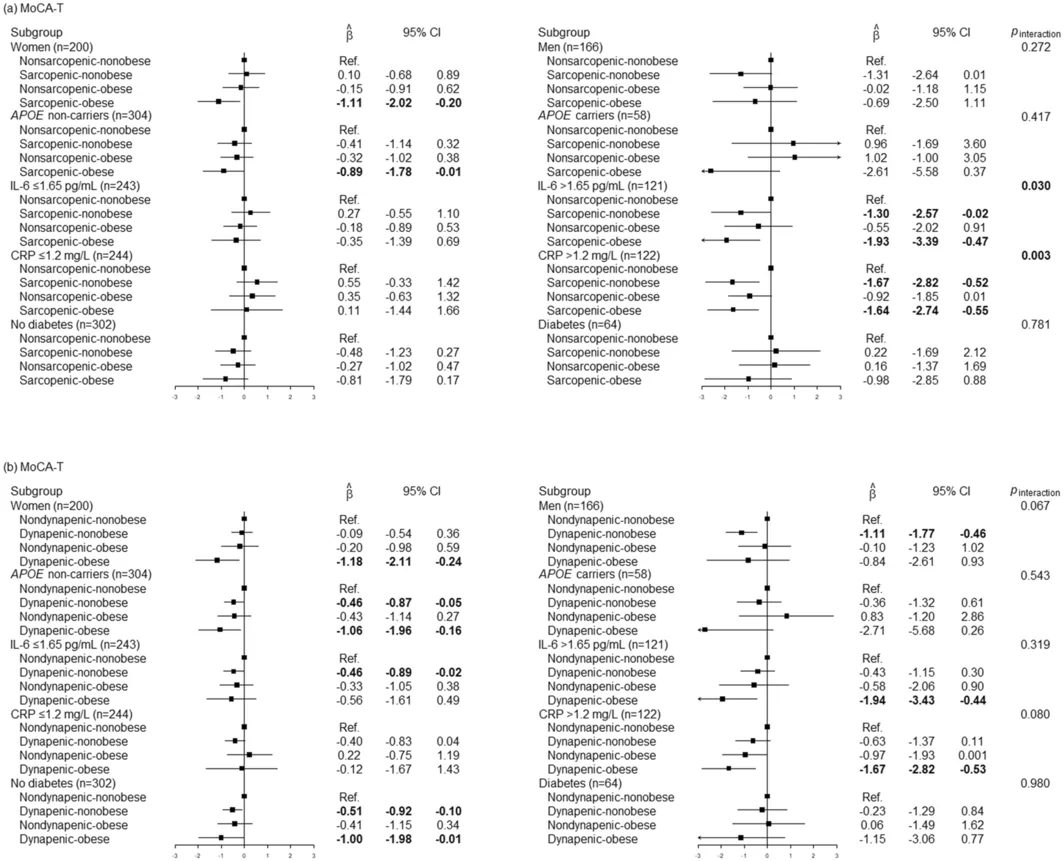
Stratified analyses conducted by multivariable‐adjusted generalized linear mixed models for the Montreal Cognitive Assessment‐Taiwanese version (MoCA‐T). (a) Participants categorized into four groups on the basis of sarcopenic obesity status. (b) Participants categorized into four groups on the basis of dynapenic obesity status.

**FIGURE 3:**
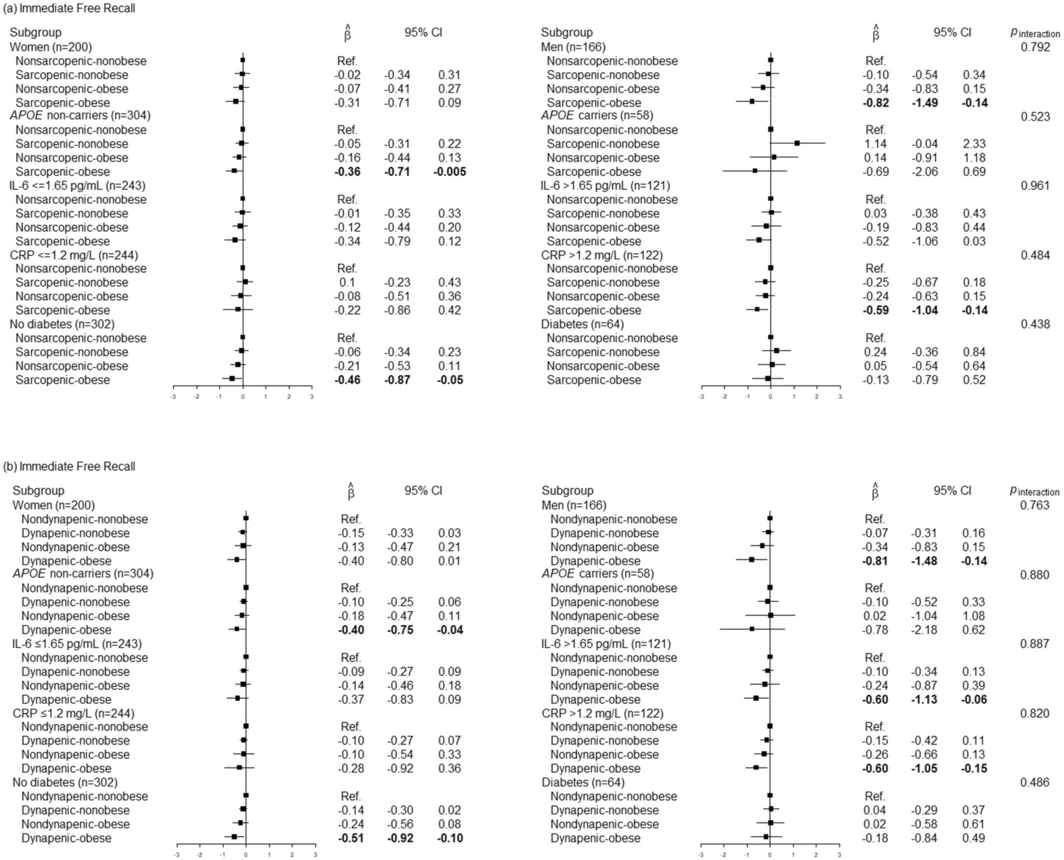
Stratified analyses conducted by multivariable‐adjusted generalized linear mixed models for the immediate free recall test. (a) Participants categorized into four groups on the basis of sarcopenic obesity status. (b) Participants categorized into four groups on the basis of dynapenic obesity status.

**FIGURE 4:**
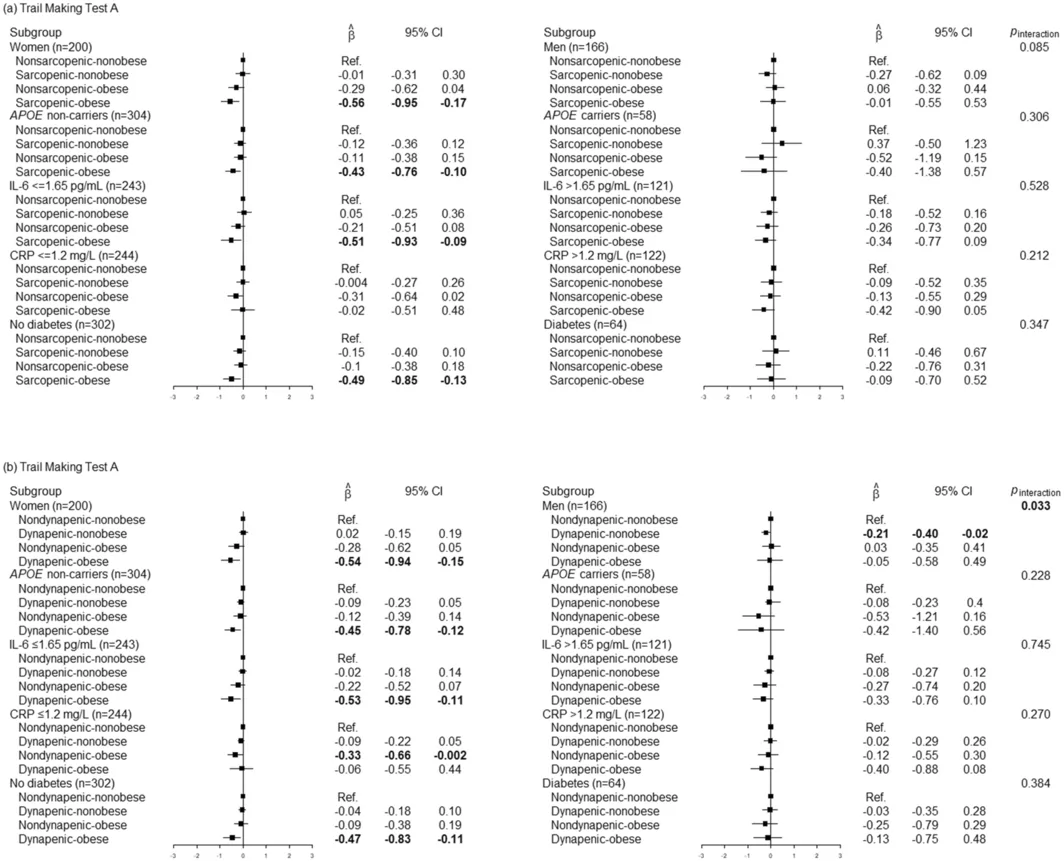
Stratified analyses conducted by multivariable‐adjusted generalized linear mixed models for the Trail Making Test A. (a) Participants categorized into four groups on the basis of sarcopenic obesity status. (b) Participants categorized into four groups on the basis of dynapenic obesity status.

Sensitivity analyses using alternative obesity definitions (waist circumference and fat mass index) yielded similar results. When waist circumference exceeded the cutoffs (≥ 90 cm for men and ≥ 80 cm for women), sarcopenic obesity was associated with poorer global cognition (MoCA‐T: $\hat{\beta }$ = − 0.68; 95% CI: −1.29 to −0.06) and attention performance (Trail Making Test A: $\hat{\beta }$ = − 0.24; 95% CI: −0.46 to −0.03) (Figure 5 and Table S2). When the highest quartile of fat mass index (≥ 6 kg/m^2^ for men and ≥ 9.26 kg/m^2^ for women) was used, sarcopenic obesity remained associated with poorer global cognition (MoCA‐T: $\hat{\beta }$ = − 0.64; 95% CI: −1.25 to −0.03) and memory (immediate free recall: $\hat{\beta }$ = − 0.26; 95% CI: −0.50 to −0.01) (Figure 5 and Table S3). When alternative sex‐specific thresholds for weight‐adjusted muscle mass (the lowest tertile and lowest quintile) were used, sarcopenic obesity remained associated with poorer global cognition, memory and attention performance (Tables S4 and S5). After the seven participants who had been excluded solely because of a BMI < 18.5 kg/m^2^ were re‐included, the expanded analytic sample (*n* = 373) yielded estimates similar to those from the original sample (*n* = 366), and the conclusions remained unchanged (Table S6).

**FIGURE 5:**
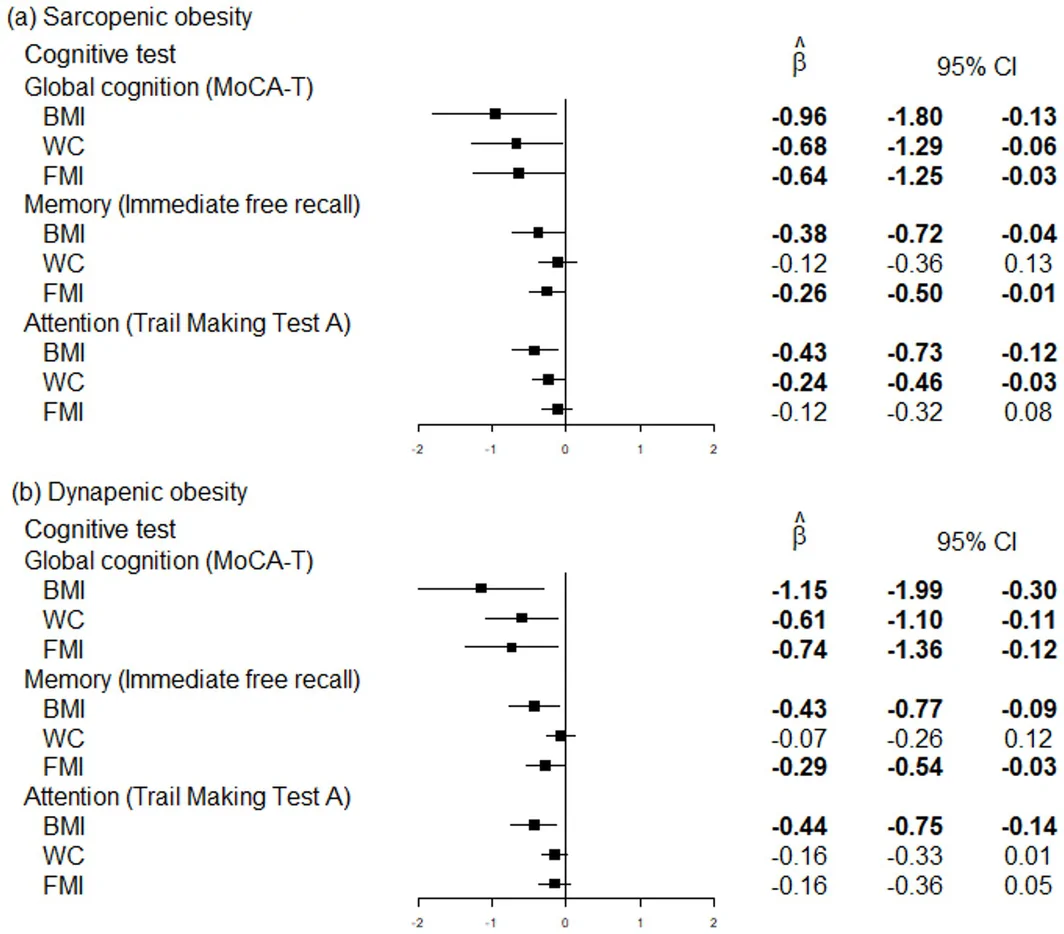
Associations of (a) sarcopenic obesity and (b) dynapenic obesity with selected cognitive outcomes across alternative obesity definitions based on body mass index (BMI; ≥ 27 kg/m^2^), waist circumference (WC; ≥ 90 cm in men and ≥ 80 cm in women) and fat mass index (FMI; highest sex‐specific quartile). Models were adjusted for the same covariates as those used in the primary analyses. Error bars represent 95% confidence intervals.

### 3.3 Dynapenic Obesity and Cognitive Function

In the multivariable analyses (Table 3), Model 1 (upper part) revealed that participants with dynapenic obesity were associated with poorer global cognition (MoCA‐T: $\hat{\beta }$ = − 1.15; 95% CI: −1.99 to −0.30), memory (immediate free recall: $\hat{\beta }$ = − 0.43; 95% CI: −0.77 to −0.09) and attention performance (Trail Making Test A: $\hat{\beta }$ = − 0.44; 95% CI: −0.75 to −0.14) compared with those without dynapenia or obesity. Dynapenia alone was associated with poorer global cognition (MoCA‐T: $\hat{\beta }$ = − 0.48; 95% CI: −0.85 to −0.10), memory (delayed free recall: $\hat{\beta }$ = − 0.18; 95% CI: −0.32 to −0.03), executive function (Trail Making Test B: $\hat{\beta }$ = − 0.17; 95% CI: −0.31 to −0.03) and attention (digit span forward: $\hat{\beta }$ = − 0.15; 95% CI: −0.30 to −0.01). Obesity alone was associated with poorer executive function, as assessed by the Trail Making Test B ($\hat{\beta }$ = − 0.34; 95% CI: −0.62 to −0.06). In Model 2 (lower part), baseline dynapenic obesity was associated with poorer global cognition, memory, attention performance and executive function. Baseline dynapenia alone was associated with poorer memory and attention. Baseline dynapenic obesity was associated with poor Trail Making Test B performance and showed a significant interaction with follow‐up time, suggesting that executive function over time was poorer in participants with baseline dynapenic obesity. Baseline dynapenia alone also showed a significant interaction with follow‐up time for the MoCA‐T, suggesting that global cognition over time was poorer in participants with baseline dynapenia alone.

**TABLE 3:** Association between dynapenic obesity status and cognitive function during 2015–2019.

Dynapenic obesity status	Global cognitive function	Logical memory				Trail making test		Digit span		Verbal fluency
	MoCA‐T $\hat{\beta }$ (95% CI)	Immediate theme recall $\hat{\beta }$ (95% CI)	Immediate free recall $\hat{\beta }$ (95% CI)	Delayed theme recall $\hat{\beta }$ (95% CI)	Delayed free recall $\hat{\beta }$ (95% CI)	A $\hat{\beta }$ (95% CI)	B $\hat{\beta }$ (95% CI)	Forward $\hat{\beta }$ (95% CI)	Backward $\hat{\beta }$ (95% CI)	$\hat{\beta }$ (95% CI)
Nondynapenic‐nonobese	Reference	Reference	Reference	Reference	Reference	Reference	Reference	Reference	Reference	Reference
Dynapenic‐nonobese	**−0.48 (−0.85, −0.10)**	−0.07 (−0.25, 0.10)	−0.11 (−0.25, 0.04)	−0.18 (−0.36, 0.001)	**−0.18 (−0.32, −0.03)**	−0.06 (−0.19, 0.06)	**−0.17 (−0.31, −0.03)**	**−0.15 (−0.30, −0.01)**	−0.05 (−0.20, 0.11)	−0.12 (−0.25, 0.02)
Nondynapenic‐obese	−0.26 (−0.91, 0.39)	−0.14 (−0.45, 0.17)	−0.16 (−0.43, 0.12)	−0.24 (−0.57, 0.08)	−0.21 (−0.49, 0.07)	−0.19 (−0.44, 0.06)	**−0.34 (−0.62, −0.06)**	−0.02 (−0.29, 0.25)	−0.03 (−0.31, 0.25)	−0.10 (−0.36, 0.17)
Dynapenic‐obese	**−1.15 (−1.99, −0.30)**	−0.26 (−0.66, 0.14)	**−0.43 (−0.77, −0.09)**	−0.16 (−0.57, 0.25)	−0.30 (−0.65, 0.05)	**−0.44 (−0.75, −0.14)**	−0.01 (−0.35, 0.32)	0.08 (−0.26, 0.41)	−0.17 (−0.53, 0.18)	−0.08 (−0.41, 0.25)
Nondynapenic‐nonobese	Reference	Reference	Reference	Reference	Reference	Reference	Reference	Reference	Reference	Reference
Dynapenic‐nonobese	−0.19 (−0.70, 0.33)	−0.03 (−0.27, 0.21)	−0.19 (−0.43, 0.04)	−0.13 (−0.38, 0.12)	**−0.27 (−0.51, −0.03)**	−0.19 (−0.40, 0.01)	−0.16 (−0.39, 0.07)	**−0.26 (−0.48, −0.05)**	−0.08 (−0.30, 0.14)	−0.12 (−0.35, 0.11)
Dynapenic‐nonobese × time	**−0.32 (−0.62, −0.01)**	−0.07 (−0.20, 0.06)	−0.04 (−0.14, 0.05)	0.03 (−0.10, 0.16)	−0.03 (−0.13, 0.07)	0.03 (−0.05, 0.12)	**0.09 (0.002, 0.19)**	−0.01 (−0.11, 0.09)	0.04 (−0.08, 0.15)	−0.08 (−0.17, 0.01)
Nondynapenic‐obese	0.03 (−0.84, 0.90)	−0.04 (−0.45, 0.37)	−0.10 (−0.50, 0.29)	−0.27 (−0.70, 0.16)	−0.25 (−0.65, 0.15)	−0.17 (−0.51, 0.18)	−0.12 (−0.51, 0.27)	0.01 (−0.36, 0.38)	−0.02 (−0.39, 0.35)	0.02 (−0.37, 0.42)
Nondynapenic‐obese × time	0.08 (−0.44, 0.61)	0.13 (−0.09, 0.36)	−0.04 (−0.20, 0.12)	**0.25 (0.03, 0.47)**	0.07 (−0.10, 0.24)	0.02 (−0.12, 0.16)	−0.11 (−0.26, 0.05)	0.14 (−0.03, 0.31)	0.11 (−0.08, 0.31)	−0.04 (−0.20, 0.11)
Dynapenic‐obese	**−1.26 (−2.38, −0.14)**	−0.42 (−0.95, 0.11)	**−0.62 (−1.13, −0.12)**	−0.29 (−0.84, 0.26)	−0.45 (−0.97, 0.07)	**−1.19 (−1.63, −0.75)**	**−0.52 (−1.02, −0.01)**	−0.10 (−0.58, 0.37)	−0.23 (−0.71, 0.24)	−0.15 (−0.66, 0.35)
Dynapenic‐obese × time	−0.48 (−1.19, 0.24)	0.08 (−0.23, 0.38)	0.003 (−0.22, 0.22)	0.04 (−0.27, 0.34)	−0.08 (−0.31, 0.15)	0.14 (−0.06, 0.34)	**−0.25 (−0.47, −0.03)**	0.13 (−0.11, 0.37)	−0.15 (−0.42, 0.11)	−0.07 (−0.28, 0.15)

*Note:* Generalized linear mixed models were used to estimate β coefficients for the cognitive variables (MoCA‐T scores or *z* scores of the cognitive domains). Adjusted for age, sex, education years, *APOE* ε4 status, depressive symptoms, follow‐up time, practice effect, cigarette smoking, alcohol consumption, hypertension, diabetes mellitus, dyslipidaemia, heart disease, physical activity by IPAQ, and interleukin‐6 and C‐reactive protein levels. The numbers in bold indicate significant findings (*p* < 0.05).

Abbreviations: CI, confidence interval; IPAQ, International Physical Activity Questionnaire; MoCA‐T, Montreal Cognitive Assessment‐Taiwanese version.

Dynapenic obesity showed similar patterns in exploratory stratified analyses. It was associated with poorer MoCA‐T performance among women, *APOE* ε4 non‐carriers, participants without diabetes and those in the highest tertile of IL‐6 or CRP ($\hat{\beta }$ = − 1.94 to −1.00; all *p* < 0.05; Figure 2); with poorer immediate free recall among men and the corresponding *APOE* ε4, diabetes and inflammatory‐marker subgroups ($\hat{\beta }$ = − 0.81 to −0.40; all *p* < 0.05; Figure 3); and with poorer Trail Making Test A performance among women, *APOE* ε4 non‐carriers, participants without diabetes and those in the lower two tertiles of IL‐6 ($\hat{\beta }$ = − 0.54 to −0.45; all *p* < 0.05; Figure 4). Dynapenia alone was associated with poorer global cognition, delayed free recall, Trail Making Test B performance and digit span forward in selected subgroups ($\hat{\beta }$ = − 1.11 to −0.18; all *p* < 0.05; data not shown).

Sensitivity analyses using alternative obesity definitions revealed that dynapenic obesity remained associated with poorer global cognitive function, whereas dynapenia alone remained associated with poorer memory and executive function (Figure 5, Tables S7 and S8). After re‐including participants who had been excluded solely because of a BMI < 18.5 kg/m^2^, the estimates were similar to those from the original analytic sample, and the conclusions remained unchanged (Table S9).

### 3.4 Muscle Mass–Strength Combinations Among Participants With Obesity

Among the 43 participants with BMI‐defined obesity at baseline, 16 had both low muscle mass and low muscle strength, 25 had low muscle mass with normal muscle strength, 2 had normal muscle mass and normal muscle strength and none had low muscle strength with normal muscle mass. Thus, sarcopenic obesity completely overlapped with dynapenic obesity in this cohort. In addition, nearly all participants with obesity met the internally defined criterion for low weight‐adjusted muscle mass. Given the resulting sparse and highly unbalanced cells, formal comparisons among the muscle mass–strength combinations were not considered statistically reliable.

## 4 Discussion

This longitudinal study of community‐dwelling older adults revealed that sarcopenic obesity was associated with poor global cognition, memory and attention performance, particularly among *APOE* ε4 non‐carriers. Importantly, this investigation employed the consensus‐defined sarcopenic obesity and applied multiple obesity definitions with ethnicity‐specific cutoffs when associations with cognitive function were examined. Our findings reinforce the growing body of evidence linking sarcopenic obesity to cognitive impairment [13, 14, 34]. We additionally examined dynapenic obesity as a related, more readily assessed strength‐based obesity phenotype with potential relevance to clinical and community screening.

Under the ESPEN–EASO framework, sarcopenic obesity is conceptually nested within dynapenic obesity. In our data, however, all participants with dynapenic obesity also fulfilled the criteria for sarcopenic obesity, precluding a direct comparison between the two phenotypes. Accordingly, our findings should not be interpreted as evidence that dynapenic obesity is equivalent to sarcopenic obesity or that muscle strength is more important than muscle mass. Rather, they suggest that a strength‐based obesity phenotype, assessed using handgrip strength and BMI, may help identify older adults with a clinically relevant muscle–adiposity imbalance who are vulnerable to poor cognitive function. Confirmation in larger cohorts with more balanced distributions of muscle mass and muscle strength is needed to determine whether dynapenic obesity provides information distinct from consensus‐defined sarcopenic obesity.

The reasons for inconsistent results across previous studies include variations in age distribution, follow‐up duration and use of different cognitive assessment tools. For example, whereas one cohort study evaluated incident dementia for more than a decade [14], our study focused on preclinical and prodromal cognitive impairment in older adults without prevalent dementia. The stronger evidence observed in our cohort may therefore reflect an association of sarcopenic obesity with earlier cognitive impairment that precedes overt dementia. Moreover, heterogeneity in the diagnostic criteria of sarcopenic obesity complicates direct comparisons of prevalence and association with cognitive performance across studies [7].

Associations between dynapenic obesity and cognitive decline have been reported in the US, Japanese and Mexican cohorts [8, 9, 35]. These findings support the potential clinical relevance of dynapenic obesity as a pragmatic screening phenotype when direct assessment of muscle mass is unavailable. However, our study did not differentiate dynapenic obesity from sarcopenic obesity with respect to cognitive impairment, and comparisons between muscle strength and muscle mass cannot be made because of the highly unbalanced combinations of muscle mass and strength. Further confirmation in larger cohorts with mutually exclusive phenotype groups is needed.

Our results also suggest potential sex‐based differences: Sarcopenic obesity and dynapenic obesity are associated with impairment in global cognition and attention performance in women. This pattern is partly consistent with a previous study from Japan, which revealed that the coexistence of decreased handgrip strength and elevated BMI was associated with mild cognitive impairment as assessed by the Montreal Cognitive Assessment in women [9]. These observations may reflect sex‐specific vulnerabilities related to muscle strength decline and differences in inflammatory and hormonal profiles [36, 37]. However, these findings should be interpreted cautiously because of small subgroup cell sizes and multiple comparisons. Further studies are needed to elucidate the underlying biological mechanisms involved.

At baseline (2015–2017), the prevalence of sarcopenic obesity in our study was 4.4%, which was comparable with the prevalence of 4.3%–5.3% reported among older Japanese adults using the ESPEN–EASO criteria [38]. When obesity was alternatively defined using waist circumference and fat mass index, the prevalence of sarcopenic obesity increased to 8.7% and 8.5%, respectively, approximating that obtained using a BMI threshold of ≥ 25 kg/m^2^. All of these estimates were lower than the reported global prevalence of approximately 11% among adults aged ≥ 60 years [5], although direct comparisons should be made with caution because of heterogeneity in study populations and operational definitions. Our cohort consisted of community‐dwelling participants (mean age of 75.7 years) who were generally healthy at baseline, which may explain the lower prevalence of this condition compared with clinical samples. As the cohort ages, their health profile is expected to converge towards that of the general older population.

We adopted the ESPEN–EASO conceptual framework, under which sarcopenic obesity requires both low muscle mass and strength in the presence of obesity. This approach differs from that used in earlier studies in which sarcopenia was defined on the basis of low muscle mass or low muscle strength alone. The ESPEN–EASO criteria do not include gait speed because of potential confounding from knee osteoarthritis, which is common among obese older adults [6]. Although the Asia–Oceania consensus definition for sarcopenic obesity adopts the AWGS 2019 criteria, which incorporate physical performance measures (e.g., 6‐m gait speed, Short Physical Performance Battery and the 5‐Times Sit‐to‐Stand Test) [39], the latest AWGS 2025 has simplified the diagnostic criteria of sarcopenia as concurrent low muscle mass and strength [27]. Therefore, applying the ESPEN–EASO conceptual framework to this population was methodologically appropriate.

Our sensitivity analyses, in which obesity was alternatively defined using waist circumference and fat mass index, revealed associations with global cognition that were consistent with those based on BMI‐defined obesity, supporting the robustness of our findings across different adiposity measures. These results also aligned with prior evidence suggesting that both central and overall adiposity might be associated with adverse cognitive outcomes when accompanied by low muscle strength or mass [8, 10]. Although the associations based on the BMI definition were generally stronger than those based on waist circumference or fat mass index, no formal tests were conducted to compare the estimates across adiposity definitions. One possible explanation is that the stricter BMI threshold of ≥ 27 kg/m^2^ resulted in the identification of a smaller group with a more pronounced muscle–adiposity phenotype, as reflected by the lower prevalence of sarcopenic obesity under this definition. This interpretation should nevertheless be considered exploratory.

The mechanisms linking sarcopenic obesity to cognitive impairment are likely multifactorial. Age‐related muscle decline and physical inactivity may promote adiposity, insulin resistance and low‐grade systemic inflammation, which may in turn impair muscle metabolism and contribute to vascular dysfunction and neuroinflammation. In our stratified analyses, sarcopenic obesity and dynapenic obesity were associated with poor global cognition, as assessed by the MoCA‐T, among participants in the upper tertile of IL‐6 or CRP. Although the concentrations of these markers were generally below commonly used clinical thresholds for overt inflammation, these findings suggest that relative differences in low‐grade inflammation may be relevant to cognitive vulnerability [40]. Both phenotypes were also associated with poor memory and attention performance among participants without diabetes. These patterns are compatible with possible inflammatory and metabolic pathways linking muscle–adiposity imbalance to cognitive health; however, given the observational design and exploratory nature of the subgroup analyses, they do not establish causal mechanisms.

From a clinical perspective, our findings may inform a pragmatic first‐step assessment based on obesity and muscle strength. Dynapenic obesity, identified using simple measures of handgrip strength and BMI, may represent a readily assessable screening phenotype to identify older adults who could benefit from further cognitive evaluation. When feasible, body composition assessment may provide additional information by characterizing the muscle mass component of the broader muscle–adiposity imbalance represented by sarcopenic obesity. However, the clinical utility of this approach requires validation in larger studies.

One of the major strengths of this study is its longitudinal design, which included repeated measurements of both sarcopenic obesity status and cognition. We applied the ESPEN–EASO conceptual framework, providing a more explicit and consistent operational definition of sarcopenic obesity. This study has several limitations. First, low muscle mass was defined using the lowest sex‐specific quartile of BIA‐estimated, weight‐adjusted muscle mass. This internally derived threshold may depend on the distribution of the study sample and may limit comparability with studies using externally validated clinical cutoffs. Furthermore, BIA provides an indirect estimate of body composition rather than a direct anatomical measurement of skeletal muscle and is influenced by hydration status and device‐specific prediction equations. Appendicular skeletal muscle mass assessed using dual‐energy X‐ray absorptiometry (DXA) or other imaging modalities was unavailable. Nevertheless, sensitivity analyses using alternative thresholds yielded similar conclusions. Second, obesity was defined primarily using BMI, which is widely used in clinical and epidemiological settings, has established ethnicity‐specific cutoffs in Taiwan and is readily applicable to community‐based screening. However, BMI is only an indirect proxy for adiposity and does not capture fat distribution or distinguish fat mass from lean mass, which may lead to the misclassification of adiposity. To address this limitation, we performed sensitivity analyses using waist circumference and fat mass index as alternative adiposity measures. The associations with global cognition were generally consistent across these definitions, supporting the robustness of our findings. Third, although participants with dementia at baseline were excluded to reduce the possibility of reverse causation, reverse causality cannot be completely ruled out in an observational study. Early cognitive impairment may have contributed to reduced physical activity, poor nutritional status, low muscle strength or changes in body composition. In addition, physical activity was assessed by the IPAQ, which may be subject to recall and reporting bias. Future studies with longer follow‐up periods and repeated assessments of cognition, nutritional status, muscle strength and body composition, together with objective measures of physical activity such as accelerometers or pedometers, are needed to clarify the temporal sequence of these relationships. Finally, although sarcopenic and dynapenic obesity were relatively uncommon in this cohort, the associations were generally consistent across the different cognitive outcomes and in the sensitivity analyses. Nevertheless, the small number of affected participants may limit the statistical power and precision for phenotype‐specific inference, particularly in stratified analyses, where subgroup cell sizes were further reduced. In addition, multiple subgroup comparisons were performed without adjustment for multiplicity, increasing the possibility of chance findings. These subgroup findings should therefore be considered exploratory and require confirmation in larger cohorts with more participants in each phenotype category.

Taken together, our findings suggest that sarcopenic obesity and a more readily assessed dynapenic obesity phenotype may represent clinically relevant conditions associated with early cognitive impairment. Handgrip strength and BMI may provide a practical first‐step, community‐based approach for identifying older adults who may benefit from further muscle mass and cognitive evaluations. Larger cohorts with longer follow‐up periods and integration of relevant biomarkers are needed to clarify the relationship between muscle–adiposity imbalance and neurocognitive health.

## Funding

This work was supported by the Ministry of Science and Technology in Taiwan (grant numbers: 100‐2314‐B‐002‐103, 101‐2314‐B‐002‐126‐MY3, 103‐2314‐B‐002‐033‐MY3, 104‐2314‐B‐002‐038‐MY3, 107‐2314‐B‐002‐186‐MY3, 107‐2314‐B‐002‐230, 108‐2314‐B‐002‐128‐MY2, 110‐2314‐B‐002‐068, 110‐2314‐B‐002‐129‐MY3, 111‐2314‐B‐002‐090‐MY3, 113‐2314‐B‐002‐188‐MY3, 113‐2621‐M‐002‐020 and 114‐2314‐B‐002‐062‐MY3). The sponsors had no role in the design, methods, participant recruitment, data collection, analysis and article preparation.

## Ethics Statement

All the authors of this manuscript comply with the guidelines of ethical authorship and of publishing in the *Journal of Cachexia, Sarcopenia and Muscle*.

## Consent

All participants provided informed consent for their participation in the Taiwan Initiatives for Geriatric Epidemiological Research (TIGER).

## Conflicts of Interest

The authors declare no conflicts of interest.

## Supporting information


**Table S1:** Characteristics of the study population by dynapenic obesity status at baseline (2015–2017).
**Table S2:** Association between sarcopenic obesity status and cognitive function, with obesity defined by waist circumference.
**Table S3:** Association between sarcopenic obesity status and cognitive function, with obesity defined by fat mass index.
**Table S4:** Association between sarcopenic obesity status and cognitive function, with low muscle mass defined as the lowest sex‐specific tertile.
**Table S5:** Association between sarcopenic obesity status and cognitive function, with low muscle mass defined as the lowest sex‐specific quintile.
**Table S6:** Association between sarcopenic obesity status and cognitive function, without exclusion of underweight participants (*n* = 373).
**Table S7:** Association between dynapenic obesity status and cognitive function, with obesity defined by waist circumference.
**Table S8:** Association between dynapenic obesity status and cognitive function, with obesity defined by fat mass index.
**Table S9:** Association between dynapenic obesity status and cognitive function, without exclusion of underweight participants (*n* = 373).

## Data Availability

The data that support the findings of this study are available on request from the corresponding author. The data are not publicly available due to privacy or ethical restrictions.

## References

[1] M. Zamboni , G. Mazzali , F. Fantin , A. Rossi , and V. Di Francesco , “Sarcopenic Obesity: A New Category of Obesity in the Elderly,” Nutrition, Metabolism, and Cardiovascular Diseases 18 (2008): 388–395. 10.1016/j.numecd.2007.10.002 18395429

[2] E. Roh and K. M. Choi , “Health Consequences of Sarcopenic Obesity: A Narrative Review,” Front Endocrinol (Lausanne) 11 (2020): 332. 10.3389/fendo.2020.00332 PMC7253580 32508753

[3] T. E. Dorner and A. Rieder , “Obesity Paradox in Elderly Patients With Cardiovascular Diseases,” International Journal of Cardiology 155 (2012): 56–65. 10.1016/j.ijcard.2011.01.076 21345498

[4] C. W. Li , K. Yu , N. Shyh‐Chang , et al., “Pathogenesis of Sarcopenia and the Relationship With Fat Mass: Descriptive Review,” Journal of Cachexia, Sarcopenia and Muscle 13 (2022): 781–794. 10.1002/jcsm.12901 PMC8977978 35106971

[5] Q. Gao , F. Mei , Y. Shang , et al., “Global Prevalence of Sarcopenic Obesity in Older Adults: A Systematic Review and Meta‐Analysis,” Clinical Nutrition 40 (2021): 4633–4641. 10.1016/j.clnu.2021.06.009 34229269

[6] L. M. Donini , L. Busetto , S. C. Bischoff , et al., “Definition and Diagnostic Criteria for Sarcopenic Obesity: ESPEN and EASO Consensus Statement,” Clinical Nutrition 41 (2022): 990–1000. 10.1016/j.clnu.2021.11.014 35227529

[7] L. M. Donini , L. Busetto , J. M. Bauer , et al., “Critical Appraisal of Definitions and Diagnostic Criteria for Sarcopenic Obesity Based on a Systematic Review,” Clinical Nutrition 39 (2020): 2368–2388. 10.1016/j.clnu.2019.11.024 31813698

[8] J. A. Batsis , C. Haudenschild , R. M. Roth , et al., “Incident Impaired Cognitive Function in Sarcopenic Obesity: Data From the National Health and Aging Trends Survey,” Journal of the American Medical Directors Association 22 (2021): 865–872. 10.1016/j.jamda.2020.09.008 PMC8269419 34248422

[9] Y. Someya , Y. Tamura , H. Kaga , et al., “Sarcopenic Obesity Is Associated With Cognitive Impairment in Community‐Dwelling Older Adults: The Bunkyo Health Study,” Clinical Nutrition 41 (2022): 1046–1051. 10.1016/j.clnu.2022.03.017 35390728

[10] U. Booranasuksakul , I. A. Macdonald , B. C. Stephan , and M. Siervo , “Body Composition, Sarcopenic Obesity, and Cognitive Function in Older Adults: Findings From the National Health and Nutrition Examination Survey (NHANES) 1999–2002 and 2011–2014,” Journal of the American Nutrition Association 43 (1999): 539–552. 10.1080/27697061.2024.2333310 38564377

[11] M. E. Levine and E. M. Crimmins , “Sarcopenic Obesity and Cognitive Functioning: The Mediating Roles of Insulin Resistance and Inflammation?,” Current Gerontology and Geriatrics Research 2012 (2012): 826398. 10.1155/2012/826398 PMC3352243 22611388

[12] H. Wang , S. Hai , Y. X. Liu , et al., “Associations Between Sarcopenic Obesity and Cognitive Impairment in Elderly Chinese Community‐Dwelling Individuals,” Journal of Nutrition, Health & Aging 23 (2019): 14–20. 10.1007/s12603-018-1088-3 PMC12280437 30569063

[13] M. I. Tolea , S. Chrisphonte , and J. E. Galvin , “Sarcopenic Obesity and Cognitive Performance,” Clinical Interventions in Aging 13 (2018): 1111–1119. 10.2147/CIA.S164113 PMC5995418 29922049

[14] J. Zhang , X. Na , Z. Li , et al., “Sarcopenic Obesity Is Part of Obesity Paradox in Dementia Development: Evidence From a Population‐Based Cohort Study,” BMC Medicine 22 (2024): 133. 10.1186/s12916-024-03357-4 PMC10960494 38520024

[15] U. Booranasuksakul , Z. Guan , I. A. Macdonald , K. Tsintzas , B. C. M. Stephan , and M. Siervo , “Sarcopenic Obesity and Brain Health: A Critical Appraisal of the Current Evidence,” Nutrition Bulletin 50 (2025): 30–43. 10.1111/nbu.12725 39799465

[16] T. C. Peng , W. L. Chen , L. W. Wu , Y. W. Chang , and T. W. Kao , “Sarcopenia and Cognitive Impairment: A Systematic Review and Meta‐Analysis,” Clinical Nutrition 39 (2020): 2695–2701. 10.1016/j.clnu.2019.12.014 31917049

[17] M. A. Beydoun , H. Beydoun , and Y. Wang , “Obesity and Central Obesity as Risk Factors for Incident Dementia and Its Subtypes: A Systematic Review and Meta‐Analysis,” Obesity Reviews 9 (2008): 204–218. 10.1111/j.1467-789X.2008.00473.x PMC4887143 18331422

[18] A. L. Fitzpatrick , L. H. Kuller , O. L. Lopez , et al., “Midlife and Late‐Life Obesity and the Risk of Dementia,” Archives of Neurology 66 (2009): 336–342. 10.1001/archneurol.2008.582 PMC3513375 19273752

[19] M. Shinohara , G. Gheni , J. Hitomi , G. Bu , and N. Sato , “APOE Genotypes Modify the Obesity Paradox in Dementia,” Journal of Neurology, Neurosurgery, and Psychiatry 94 (2023): 670–680. 10.1136/jnnp-2022-331034 PMC10695687 37414536

[20] A. Ionescu‐Tucker and C. W. Cotman , “Emerging Roles of Oxidative Stress in Brain Aging and Alzheimer's Disease,” Neurobiology of Aging 107 (2021): 86–95. 10.1016/j.neurobiolaging.2021.07.014 34416493

[21] H. M. Noh , S. Oh , H. J. Song , et al., “Relationships Between Cognitive Function and Body Composition Among Community‐Dwelling Older Adults: A Cross‐Sectional Study,” BMC Geriatrics 17 (2017): 1–9. 10.1186/s12877-017-0651-9 PMC5667483 29096612

[22] P. C. Yu , C. C. Hsu , W. J. Lee , et al., “Muscle‐To‐Fat Ratio Identifies Functional Impairments and Cardiometabolic Risk and Predicts Outcomes: Biomarkers of Sarcopenic Obesity,” Journal of Cachexia, Sarcopenia and Muscle 13 (2022): 368–376. 10.1002/jcsm.12877 PMC8818605 34866342

[23] N. X. Tou , S. L. Wee , B. W. J. Pang , et al., “Associations of Fat Mass and Muscle Function but Not Lean Mass With Cognitive Impairment: The Yishun Study,” PLoS ONE 16 (2021): e0256702. 10.1371/journal.pone.0256702 PMC8389410 34437646

[24] M. Yang , X. Ding , L. Luo , Q. Hao , and B. Dong , “Disability Associated With Obesity, Dynapenia and Dynapenic‐Obesity in Chinese Older Adults,” Journal of the American Medical Directors Association 15 (2014): 150.e11–150.e16. 10.1016/j.jamda.2013.10.009 24291347

[25] I. Janssen , S. B. Heymsfield , and R. Ross , “Low Relative Skeletal Muscle Mass (Sarcopenia) in Older Persons Is Associated With Functional Impairment and Physical Disability,” Journal of the American Geriatrics Society 50 (2002): 889–896. 10.1046/j.1532-5415.2002.50216.x 12028177

[26] T. C. Peng , J. M. Chiou , T. F. Chen , Y. C. Chen , and J. H. Chen , “Grip Strength and Sarcopenia Predict 2‐Year Cognitive Impairment in Community‐Dwelling Older Adults,” Journal of the American Medical Directors Association 24 (2023): 292–298. 10.1016/j.jamda.2022.10.015 36435272

[27] L. K. Chen , F. Y. Hsiao , M. Akishita , et al., “A Focus Shift From Sarcopenia to Muscle Health in the Asian Working Group for Sarcopenia 2025 Consensus Update,” Nature Aging 5 (2025): 2164–2175. 10.1038/s43587-025-01004-y 41188603

[28] Department of Health , “Identification, Evaluation, and Treatment of Overweight and Obesity in Adults in Taiwan,” (2003).

[29] World Health Organization , The Asia‐Pacific Perspective: Redefining Obesity and Its Treatment (Health Communications Australia, 2000).

[30] P. Liu , F. Ma , H. Lou , and Y. Liu , “The Utility of Fat Mass Index Vs. Body Mass Index and Percentage of Body Fat in the Screening of Metabolic Syndrome,” BMC Public Health 13 (2013): 629. 10.1186/1471-2458-13-629 PMC3703297 23819808

[31] Y. H. Cheng , S. C. Lee , Y. C. Chen , et al., “Normative Study of the Taiwanese Version of the Montreal Cognitive Assessment (MoCA) in Community‐Dwelling Individuals in Taiwan,” Journal of the Chinese Medical Association 88 (2025): 617–623. 10.1097/JCMA.0000000000001265 PMC12637103 40626349

[32] Y. M. Liou , C. J. Jwo , K. G. Yao , L. C. Chiang , and L. H. Huang , “Selection of Appropriate Chinese Terms to Represent Intensity and Types of Physical Activity Terms for Use in the Taiwan Version of IPAQ,” Journal of Nursing Research: JNR 16 (2008): 252–263. 10.1097/01.jnr.0000387313.20386.0a 19061172

[33] Y. C. Chou , M. S. Lee , J. M. Chiou , T. F. Chen , Y. C. Chen , and J. H. Chen , “Association of Diet Quality and Vegetable Variety With the Risk of Cognitive Decline in Chinese Older Adults,” Nutrients 11 (2019): 1666. 10.3390/nu11071666 PMC6682985 31330854

[34] U. Booranasuksakul , K. Tsintzas , I. Macdonald , B. C. M. Stephan , and M. Siervo , “Application of a New Definition of Sarcopenic Obesity in Middle‐Aged and Older Adults and Association With Cognitive Function: Findings From the National Health and Nutrition Examination Survey 1999–2002,” Clinical Nutrition ESPEN 63 (2024): 919–928. 10.1016/j.clnesp.2024.08.017 39181532

[35] R. H. Ju and S. Al Snih , “The Role of Dynapenia and Obesity on Cognitive Function in Older Adults,” Clinical Nutrition ESPEN 63 (2024): 191–196. 10.1016/j.clnesp.2024.06.039 PMC11424253 38963765

[36] D. T. A. Vithran , M. H. Y. Hassan , M. Rahmati , et al., “Sex‐Stratified Handgrip Strength and Adipose‐Derived Inflammatory Biomarkers in Sarcopenia—A Systematic Review and Meta‐Analysis,” BMC Geriatrics 26 (2025): 53. 10.1186/s12877-025-06777-w PMC12801484 41366336

[37] A. J. Critchlow , S. E. Alexander , D. S. Hiam , L. Ferrucci , D. Scott , and S. Lamon , “Associations Between Female Sex Hormones and Skeletal Muscle Ageing: The Baltimore Longitudinal Study of Aging,” Journal of Cachexia, Sarcopenia and Muscle 16 (2025): e13786. 10.1002/jcsm.13786 PMC12037696 40296368

[38] A. Shimizu , K. Maeda , J. Ueshima , et al., “Prevalence of Sarcopenic Obesity Based on Newly Proposed Diagnostic Criteria and Functional Outcomes in Older Adults Undergoing Rehabilitation,” Mechanisms of Ageing and Development 208 (2022): 111728. 10.1016/j.mad.2022.111728 36084796

[39] T. P. Chen , H. H. Kao , W. Ogawa , et al., “The Asia–Oceania Consensus: Definitions and Diagnostic Criteria for Sarcopenic Obesity,” Obesity Research & Clinical Practice 19 (2025): 185–192. 10.1016/j.orcp.2025.05.001 40335420

[40] K. Yaffe , A. Kanaya , K. Lindquist , et al., “The Metabolic Syndrome, Inflammation, and Risk of Cognitive Decline,” Journal of the American Medical Association 292 (2004): 2237–2242. 10.1001/jama.292.18.2237 15536110

