# Supplementary material for: Consensus‐Defined Sarcopenic Obesity and a Related Dynapenic Obesity Phenotype in Relation to Early Cognitive Impairment
Source: J Cachexia Sarcopenia Muscle. 2026 Oct 2;17(5):e70395. doi: 10.1002/jcsm.70395 (PMC13632612; doi:10.1002/jcsm.70395)
Supplement: Supplementary file 1 [file JCSM-17-e70395-s001.docx]

**SUPPLEMENTARY TABLE 1**	Characteristics of the study population by dynapenic obesity status at baseline (2015‒2017)

| Variables | Missing  *n* | Total  (*n*=366) | Nondynapenic-nonobese  (*n*=204) | Dynapenic-nonobese  (*n*=119) | Nondynapenic-obese  (*n*=27) | Dynapenic-obese  (*n*=16) | *p* value |
| --- | --- | --- | --- | --- | --- | --- | --- |
|  |  | Median (IQR) | | | | |  |
| Age (year) | 0 | 74.8 (71.8, 78.7) | 74.2 (71.6, 77.1) | 76.9 (72.4, 81.9) | 72.9 (70.6, 76.2) | 76.2 (71.4, 79.4) | **<0.001** |
| Education (year) | 0 | 14.0 (12.0, 16.0) | 15.0 (12.0, 16.0) | 14.0 (12.0, 16.0) | 14.0 (11.0, 16.0) | 12.0 (9.0, 16.0) | 0.059 |
| Physical activity  (1000 MET-min/week) | 0 | 1.5 (0.8, 3.0) | 1.9 (0.9, 3.2) | 1.4 (0.8, 2.5) | 2.8 (0.7, 4.0) | 0.9 (0.1, 1.3) | **0.004** |
| ADL score | 2 | 100 (100, 100) | 100 (100, 100) | 100 (100, 100) | 100 (100, 100) | 100 (100, 100) | **0.037** |
| IADL score | 2 | 8 (8, 8) | 8 (8, 8) | 8 (8, 8) | 8 (8, 8) | 8 (8, 8) | 0.082 |
| mAHEI score | 6 | 37.6 (31.7, 42.8) | 38.2 (31.8, 42.6) | 37.4 (32.5, 43.0) | 36.4 (26.9, 43.8) | 36.1 (25.5, 39.4) | 0.422 |
| Gait speed (m/s) | 0 | 0.8 (0.6, 0.9) | 0.8 (0.7, 0.9) | 0.7 (0.6, 0.8) | 0.8 (0.7, 0.9) | 0.6 (0.6, 0.7) | **<0.001** |
| Handgrip strength (kg) | 0 | 22.0 (18.0, 30.0) | 28.0 (20.8, 32.0) | 16.0 (14.0, 23.0) | 26.0 (20.0, 33.5) | 16.0 (14.0, 16.8) | **<0.001** |
| Weight-adjusted total muscle mass (kg/kg) | 0 | 0.7 (0.6, 0.7) | 0.7 (0.6, 0.8) | 0.7 (0.6, 0.7) | 0.6 (0.5, 0.7) | 0.6 (0.5, 0.6) | **<0.001** |
| Fat mass (%) | 0 | 27.4 (21.0, 33.1) | 25.0 (19.8, 31.6) | 28.2 (21.0, 33.0) | 38.7 (26.7, 42.8) | 39.9 (39.1, 43.8) | **<0.001** |
| Fat mass index (kg/m^2^) | 0 | 6.3 (4.8, 7.9) | 5.8 (4.6, 7.2) | 6.0 (4.8, 7.8) | 10.6 (7.5, 12.7) | 11.1 (10.7, 13.2) | **<0.001** |
| BMI (kg/m^2^) | 0 | 23.4 (21.6, 25.4) | 23.2 (21.4, 24.7) | 22.8 (21.2, 24.6) | 28.3 (27.5, 31.0) | 28.6 (27.7, 31.1) | **<0.001** |
| Waist circumference (cm) | 0 | 84.3 (78.0, 91.0) | 83.0 (77.3, 89.0) | 83.0 (77.0, 88.0) | 95.0 (92.0, 99.0) | 94.0 (90.0, 102.3) | **<0.001** |
| C-reactive protein (mg/L) | 0 | 0.6 (0.3, 1.7) | 0.6 (0.3, 1.5) | 0.6 (0.4, 1.7) | 1.5 (0.6, 3.2) | 2.0 (1.2, 2.7) | **<0.001** |
| Interleukin-6 (pg/mL) | 2 | 1.5 (1.2, 1.8) | 1.4 (1.2, 1.8) | 1.5 (1.3, 1.8) | 1.4 (1.2, 1.7) | 1.4 (1.3, 1.8) | 0.663 |
|  |  | *n* (%) | | | | |  |
| Women | 0 | 200 (54.6) | 98 (48.0) | 73 (61.3) | 16 (59.3) | 13 (81.3) | **0.014** |
| Annual household income > 1 million (NTD) | 9 | 75 (21.0) | 44 (21.9) | 23 (20.0) | 6 (24.0) | 2 (12.5) | 0.843 |
| Cigarette smoking | 0 | 49 (13.4) | 32 (15.7) | 13 (10.9) | 3 (11.1) | 1 (6.3) | 0.597 |
| Alcohol consumption | 0 | 48 (13.1) | 30 (14.7) | 12 (10.1) | 4 (14.8) | 2 (12.5) | 0.643 |
| *APOE* ε4 carriers | 4 | 58 (16.0) | 36 (17.8) | 17 (14.5) | 3 (11.1) | 2 (12.5) | 0.808 |
| Depressive symptoms | 0 | 25 (6.8) | 9 (4.4) | 12 (10.1) | 1 (3.7) | 3 (18.8) | **0.041** |
| Hypertension | 0 | 230 (62.8) | 123 (60.3) | 78 (65.6) | 18 (66.7) | 11 (68.8) | 0.718 |
| Hyperlipidemia | 0 | 165 (45.1) | 91 (44.6) | 50 (42.0) | 17 (63.0) | 7 (43.8) | 0.265 |
| Diabetes mellitus | 0 | 64 (17.5) | 28 (13.7) | 21 (17.7) | 9 (33.3) | 6 (37.5) | **0.012** |
| Heart disease | 0 | 129 (35.3) | 62 (30.4) | 50 (42.0) | 8 (29.6) | 9 (56.3) | **0.047** |

*Note:* *P* values were calculated by the Kruskal‒Wallis test for continuous variables and the chi‒square test (or Fisher’s exact test as appropriate) for categorical variables. The numbers in bold indicate significant findings (*p* < 0.05).

Abbreviations: MET, metabolic equivalent of task; ADL, Activities of Daily Living; IADL, Instrumental Activities of Daily Living; mAHEI, modified Alternative Healthy Eating Index; BMI, body mass index; NTD, new Taiwan dollar; *APOE*, apolipoprotein E; IQR, interquartile range.

**SUPPLEMENTARY TABLE 2**	Association between sarcopenic obesity status and cognitive function, with obesity defined by waist circumference

| **Cognitive**  **function**  **Sarcopenic**  **obesity**  **status** |  | **Global cognitive function** |  | **Logical memory** | | | |  | **Trail Making Test** | |  | **Digit span** | |  | **Verbal fluency** |
| --- | --- | --- | --- | --- | --- | --- | --- | --- | --- | --- | --- | --- | --- | --- | --- |
|  |  | MoCA-T  $\hat{\beta}$ (95% CI) |  | Immediate  theme recall  $\hat{\beta}$ (95% CI) | Immediate  free recall  $\hat{\beta}$ (95% CI) | Delayed  theme recall  $\hat{\beta}$ (95% CI) | Delayed  free recall  $\hat{\beta}$ (95% CI) |  | A  $\hat{\beta}$ (95% CI) | B  $\hat{\beta}$ (95% CI) |  | Forward  $\hat{\beta}$ (95% CI) | Backward  $\hat{\beta}$ (95% CI) |  | $\hat{\beta}$ (95% CI) |
| Nonsarcopenic-nonobese |  | Reference |  | Reference | Reference | Reference | Reference |  | Reference | Reference |  | Reference | Reference |  | Reference |
| Sarcopenic-nonobese |  | 0.78  (-0.96, 2.52) |  | 0.34  (-0.46, 1.14) | 0.28  (-0.34, 0.90) | 0.35  (-0.46, 1.16) | 0.18  (-0.46, 0.82) |  | 0.10  (-0.45, 0.65) | -0.09  (-0.71, 0.52) |  | -0.51  (-1.15, 0.13) | -0.11  (-0.81, 0.58) |  | 0.35  (-0.24, 0.94) |
| Nonsarcopenic-obese |  | 0.04  (-0.31, 0.39) |  | 0.02  (-0.14, 0.19) | 0.06  (-0.08, 0.19) | -0.04  (-0.20, 0.13) | 0.05  (-0.10, 0.19) |  | -0.06  (-0.18, 0.06) | -0.09  (-0.22, 0.05) |  | 0.05  (-0.09, 0.19) | -0.05  (-0.19, 0.10) |  | -0.07  (-0.20, 0.06) |
| Sarcopenic-obese |  | **-0.68**  **(-1.29, -0.06)** |  | 0.12  (-0.17, 0.41) | -0.12  (-0.36, 0.13) | 0.01  (-0.29, 0.31) | -0.01  (-0.26, 0.25) |  | **-0.24**  **(-0.46, -0.03)** | 0.04  (-0.21, 0.28) |  | 0.03  (-0.22, 0.27) | -0.13  (-0.39, 0.13) |  | -0.09  (-0.33, 0.14) |

*Note*: Generalized linear mixed models were used to estimate $\beta$ coefficients for the cognitive variables (MoCA-T scores or z scores of the cognitive domains). Adjusted for age, sex, education years, *APOE* ε4 status, depressive symptoms, follow-up time, practice effect, cigarette smoking, alcohol consumption, hypertension, diabetes mellitus, dyslipidemia, heart disease, physical activity by IPAQ, and interleukin-6 and C-reactive protein levels. The numbers in bold indicate significant findings (*p* < 0.05).

Abbreviations: MoCA-T, Montreal Cognitive Assessment-Taiwanese version; IPAQ, International Physical Activity Questionnaire.

**SUPPLEMENTARY TABLE 3**	Association between sarcopenic obesity status and cognitive function, with obesity defined by fat mass index

| **Cognitive**  **function**  **Sarcopenic**  **obesity**  **status** |  | **Global cognitive function** |  | **Logical memory** | | | |  | **Trail Making Test** | |  | **Digit span** | |  | **Verbal fluency** |
| --- | --- | --- | --- | --- | --- | --- | --- | --- | --- | --- | --- | --- | --- | --- | --- |
|  |  | MoCA-T  $\hat{\beta}$ (95% CI) |  | Immediate  theme recall  $\hat{\beta}$ (95% CI) | Immediate  free recall  $\hat{\beta}$ (95% CI) | Delayed  theme recall  $\hat{\beta}$ (95% CI) | Delayed  free recall  $\hat{\beta}$ (95% CI) |  | A  $\hat{\beta}$ (95% CI) | B  $\hat{\beta}$ (95% CI) |  | Forward  $\hat{\beta}$ (95% CI) | Backward  $\hat{\beta}$ (95% CI) |  | $\hat{\beta}$ (95% CI) |
| Nonsarcopenic-nonobese |  | Reference |  | Reference | Reference | Reference | Reference |  | Reference | Reference |  | Reference | Reference |  | Reference |
| Sarcopenic-nonobese |  | -0.15  (-1.68, 1.37) |  | 0.62  (-0.09, 1.32) | 0.41  (-0.14, 0.96) | 0.10  (-0.62, 0.82) | 0.24  (-0.33, 0.82) |  | 0.02  (-0.44, 0.48) | -0.24  (-0.78, 0.31) |  | 0.32  (-0.25, 0.89) | -0.05  (-0.67, 0.56) |  | 0.36  (-0.17, 0.89) |
| Nonsarcopenic-obese |  | 0.25  (-0.24, 0.73) |  | -0.10  (-0.33, 0.13) | -0.10  (-0.31, 0.11) | -0.15  (-0.39, 0.09) | -0.07  (-0.28, 0.15) |  | -0.08  (-0.24, 0.09) | -0.09  (-0.30, 0.12) |  | 0.02  (-0.18, 0.23) | 0.04  (-0.17, 0.24) |  | 0.04  (-0.17, 0.25) |
| Sarcopenic-obese |  | **-0.64**  **(-1.25, -0.03)** |  | 0.003  (-0.29, 0.29) | **-0.26**  **(-0.50, -0.01)** | -0.02  (-0.32, 0.28) | -0.11  (-0.36, 0.15) |  | -0.12  (-0.32, 0.08) | 0.14  (-0.11, 0.39) |  | -0.13  (-0.38, 0.12) | -0.07  (-0.33, 0.19) |  | -0.05  (-0.29, 0.20) |

*Note*: Generalized linear mixed models were used to estimate $\beta$ coefficients for the cognitive variables (MoCA-T scores or z scores of the cognitive domains). Adjusted for age, sex, education years, *APOE* ε4 status, depressive symptoms, follow-up time, practice effect, cigarette smoking, alcohol consumption, hypertension, diabetes mellitus, dyslipidemia, heart disease, physical activity by IPAQ, and interleukin-6 and C-reactive protein levels. The numbers in bold indicate significant findings (*p* < 0.05).

Abbreviations: MoCA-T, Montreal Cognitive Assessment-Taiwanese version; IPAQ, International Physical Activity Questionnaire.

**SUPPLEMENTARY TABLE 4**	Association between sarcopenic obesity status and cognitive function, with low muscle mass defined as the lowest sex-specific tertile

| **Cognitive**  **function**  **Sarcopenic**  **obesity**  **status** |  | **Global cognitive function** |  | **Logical memory** | | | |  | **Trail Making Test** | |  | **Digit span** | |  | **Verbal fluency** |
| --- | --- | --- | --- | --- | --- | --- | --- | --- | --- | --- | --- | --- | --- | --- | --- |
|  |  | MoCA-T  $\hat{\beta}$ (95% CI) |  | Immediate  theme recall  $\hat{\beta}$ (95% CI) | Immediate  free recall  $\hat{\beta}$ (95% CI) | Delayed  theme recall  $\hat{\beta}$ (95% CI) | Delayed  free recall  $\hat{\beta}$ (95% CI) |  | A  $\hat{\beta}$ (95% CI) | B  $\hat{\beta}$ (95% CI) |  | Forward  $\hat{\beta}$ (95% CI) | Backward  $\hat{\beta}$ (95% CI) |  | $\hat{\beta}$ (95% CI) |
| Nonsarcopenic-nonobese |  | Reference |  | Reference | Reference | Reference | Reference |  | Reference | Reference |  | Reference | Reference |  | Reference |
| Sarcopenic-nonobese |  | -0.37  (-0.95, 0.21) |  | 0.21  (-0.05, 0.48) | 0.02  (-0.19, 0.23) | 0.11  (-0.17, 0.38) | 0.03  (-0.19, 0.25) |  | -0.03  (-0.22, 0.16) | 0.01  (-0.20, 0.22) |  | -0.07  (-0.29, 0.15) | -0.03  (-0.27, 0.20) |  | -0.08  (-0.28, 0.13) |
| Nonsarcopenic-obese |  | -0.16  (-0.81, 0.49) |  | -0.09  (-0.40, 0.22) | -0.12  (-0.39, 0.15) | -0.18  (-0.49, 0.14) | -0.15  (-0.43, 0.13) |  | -0.17  (-0.42, 0.07) | **-0.29**  **(-0.56, -0.01)** |  | 0.01  (-0.26, 0.28) | -0.02  (-0.29, 0.26) |  | -0.07  (-0.34, 0.20) |
| Sarcopenic-obese |  | **-0.98**  **(-1.81, -0.14)** |  | -0.18  (-0.58, 0.21) | **-0.37**  **(-0.71, -0.03)** | -0.05  (-0.46, 0.35) | -0.21  (-0.55, 0.14) |  | **-0.42**  **(-0.72, -0.11)** | 0.07  (-0.26, 0.41) |  | 0.13  (-0.20, 0.47) | -0.16  (-0.51, 0.19) |  | -0.05  (-0.38, 0.28) |

*Note*: Generalized linear mixed models were used to estimate $\beta$ coefficients for the cognitive variables (MoCA-T scores or z scores of the cognitive domains). Adjusted for age, sex, education years, *APOE* ε4 status, depressive symptoms, follow-up time, practice effect, cigarette smoking, alcohol consumption, hypertension, diabetes mellitus, dyslipidemia, heart disease, physical activity by IPAQ, and interleukin-6 and C-reactive protein levels. The numbers in bold indicate significant findings (*p* < 0.05).

Abbreviations: MoCA-T, Montreal Cognitive Assessment-Taiwanese version; IPAQ, International Physical Activity Questionnaire.

**SUPPLEMENTARY TABLE 5**	Association between sarcopenic obesity status and cognitive function, with low muscle mass defined as the lowest sex-specific quintile

| **Cognitive**  **function**  **Sarcopenic**  **obesity**  **status** |  | **Global cognitive function** |  | **Logical memory** | | | |  | **Trail Making Test** | |  | **Digit span** | |  | **Verbal fluency** |
| --- | --- | --- | --- | --- | --- | --- | --- | --- | --- | --- | --- | --- | --- | --- | --- |
|  |  | MoCA-T  $\hat{\beta}$ (95% CI) |  | Immediate  theme recall  $\hat{\beta}$ (95% CI) | Immediate  free recall  $\hat{\beta}$ (95% CI) | Delayed  theme recall  $\hat{\beta}$ (95% CI) | Delayed  free recall  $\hat{\beta}$ (95% CI) |  | A  $\hat{\beta}$ (95% CI) | B  $\hat{\beta}$ (95% CI) |  | Forward  $\hat{\beta}$ (95% CI) | Backward  $\hat{\beta}$ (95% CI) |  | $\hat{\beta}$ (95% CI) |
| Nonsarcopenic-nonobese |  | Reference |  | Reference | Reference | Reference | Reference |  | Reference | Reference |  | Reference | Reference |  | Reference |
| Sarcopenic-nonobese |  | -0.70  (-1.67, 0.27) |  | 0.15  (-0.30, 0.60) | 0.03  (-0.33, 0.38) | -0.19  (-0.64, 0.27) | -0.05  (-0.42, 0.31) |  | -0.11  (-0.42, 0.21) | 0.11  (-0.24, 0.46) |  | -0.15  (-0.52, 0.21) | -0.19  (-0.58, 0.20) |  | -0.19  (-0.53, 0.15) |
| Nonsarcopenic-obese |  | -0.13  (-0.76, 0.51) |  | -0.07  (-0.37, 0.24) | -0.12  (-0.39, 0.15) | -0.17  (-0.48, 0.15) | -0.17  (-0.45, 0.11) |  | -0.20  (-0.44, 0.05) | -0.27  (-0.54, 0.01) |  | 0.05  (-0.21, 0.32) | -0.02  (-0.29, 0.25) |  | -0.09  (-0.35, 0.18) |
| Sarcopenic-obese |  | **-1.08**  **(-1.94, -0.21)** |  | -0.31  (-0.72, 0.10) | **-0.39**  **(-0.73, -0.04)** | -0.14  (-0.56, 0.28) | -0.20  (-0.56, 0.16) |  | **-0.39**  **(-0.71, -0.08)** | 0.08  (-0.27, 0.43) |  | 0.06  (-0.28, 0.41) | -0.18  (-0.54, 0.18) |  | -0.01  (-0.35, 0.32) |

*Note*: Generalized linear mixed models were used to estimate $\beta$ coefficients for the cognitive variables (MoCA-T scores or z scores of the cognitive domains). Adjusted for age, sex, education years, *APOE* ε4 status, depressive symptoms, follow-up time, practice effect, cigarette smoking, alcohol consumption, hypertension, diabetes mellitus, dyslipidemia, heart disease, physical activity by IPAQ, and interleukin-6 and C-reactive protein levels. The numbers in bold indicate significant findings (*p* < 0.05).

Abbreviations: MoCA-T, Montreal Cognitive Assessment-Taiwanese version; IPAQ, International Physical Activity Questionnaire.

**SUPPLEMENTARY TABLE 6**	Association between sarcopenic obesity status and cognitive function, without exclusion of underweight participants (*n* = 373)

| **Cognitive**  **function**  **Sarcopenic**  **obesity**  **status** |  | **Global cognitive function** |  | **Logical memory** | | | |  | **Trail Making Test** | |  | **Digit span** | |  | **Verbal fluency** |
| --- | --- | --- | --- | --- | --- | --- | --- | --- | --- | --- | --- | --- | --- | --- | --- |
|  |  | MoCA-T  $\hat{\beta}$ (95% CI) |  | Immediate  theme recall  $\hat{\beta}$ (95% CI) | Immediate  free recall  $\hat{\beta}$ (95% CI) | Delayed  theme recall  $\hat{\beta}$ (95% CI) | Delayed  free recall  $\hat{\beta}$ (95% CI) |  | A  $\hat{\beta}$ (95% CI) | B  $\hat{\beta}$ (95% CI) |  | Forward  $\hat{\beta}$ (95% CI) | Backward  $\hat{\beta}$ (95% CI) |  | $\hat{\beta}$ (95% CI) |
| Nonsarcopenic-nonobese |  | Reference |  | Reference | Reference | Reference | Reference |  | Reference | Reference |  | Reference | Reference |  | Reference |
| Sarcopenic-nonobese |  | -0.30  (-0.98, 0.38) |  | 0.30  (-0.02, 0.61) | 0.01  (-0.24, 0.26) | 0.11  (-0.21, 0.44) | 0.05  (-0.21, 0.31) |  | -0.09  (-0.32, 0.13) | -0.01  (-0.25, 0.24) |  | -0.18  (-0.43, 0.08) | -0.05  (-0.33, 0.22) |  | 0.02  (-0.23, 0.26) |
| Nonsarcopenic-obese |  | -0.15  (-0.79, 0.49) |  | -0.10  (-0.40, 0.21) | -0.13  (-0.40, 0.15) | -0.18  (-0.50, 0.13) | -0.16  (-0.44, 0.12) |  | -0.18  (-0.43, 0.06) | **-0.29**  **(-0.56, -0.02)** |  | 0.01  (-0.26, 0.27) | -0.01  (-0.28, 0.27) |  | -0.06  (-0.33, 0.21) |
| Sarcopenic-obese |  | **-0.96**  **(-1.79, -0.13)** |  | -0.18  (-0.57, 0.21) | **-0.37**  **(-0.71, -0.04)** | -0.06  (-0.46, 0.35) | -0.21  (-0.55, 0.14) |  | **-0.43**  **(-0.74, -0.13)** | 0.08  (-0.26, 0.41) |  | 0.12  (-0.22, 0.45) | -0.15  (-0.50, 0.20) |  | -0.02  (-0.35, 0.31) |

*Note*: Generalized linear mixed models were used to estimate $\beta$ coefficients for the cognitive variables (MoCA-T scores or z scores of the cognitive domains). Adjusted for age, sex, education years, *APOE* ε4 status, depressive symptoms, follow-up time, practice effect, cigarette smoking, alcohol consumption, hypertension, diabetes mellitus, dyslipidemia, heart disease, physical activity by IPAQ, and interleukin-6 and C-reactive protein levels. The numbers in bold indicate significant findings (*p* < 0.05).

Abbreviations: MoCA-T, Montreal Cognitive Assessment-Taiwanese version; IPAQ, International Physical Activity Questionnaire.

**SUPPLEMENTARY TABLE 7**	Association between dynapenic obesity status and cognitive function, with obesity defined by waist circumference

| **Cognitive**  **function**  **Dynapenic**  **obesity**  **status** |  | **Global cognitive function** |  | **Logical memory** | | | |  | **Trail Making Test** | |  | **Digit span** | |  | **Verbal fluency** |
| --- | --- | --- | --- | --- | --- | --- | --- | --- | --- | --- | --- | --- | --- | --- | --- |
|  |  | MoCA-T  $\hat{\beta}$ (95% CI) |  | Immediate  theme recall  $\hat{\beta}$ (95% CI) | Immediate  free recall  $\hat{\beta}$ (95% CI) | Delayed  theme recall  $\hat{\beta}$ (95% CI) | Delayed  free recall  $\hat{\beta}$ (95% CI) |  | A  $\hat{\beta}$ (95% CI) | B  $\hat{\beta}$ (95% CI) |  | Forward  $\hat{\beta}$ (95% CI) | Backward  $\hat{\beta}$ (95% CI) |  | $\hat{\beta}$ (95% CI) |
| Nondynapenic-nonobese |  | Reference |  | Reference | Reference | Reference | Reference |  | Reference | Reference |  | Reference | Reference |  | Reference |
| Dynapenic-nonobese |  | -0.40  (-0.89, 0.09) |  | -0.17  (-0.40, 0.06) | **-0.19**  **(-0.37, -0.005)** | **-0.33**  **(-0.56, -0.10)** | **-0.36**  **(-0.55, -0.17)** |  | -0.06  (-0.22, 0.10) | **-0.23**  **(-0.41, -0.04)** |  | -0.06  (-0.25, 0.12) | -0.05  (-0.25, 0.15) |  | -0.05  (-0.23, 0.12) |
| Nondynapenic-obese |  | 0.01  (-0.40, 0.43) |  | -0.04  (-0.23, 0.16) | -0.01  (-0.17, 0.16) | -0.17  (-0.37, 0.03) | -0.10  (-0.26, 0.07) |  | -0.06  (-0.21, 0.08) | -0.15  (-0.31, 0.01) |  | 0.10  (-0.06, 0.26) | -0.04  (-0.21, 0.13) |  | -0.04  (-0.19, 0.11) |
| Dynapenic-obese |  | **-0.61**  **(-1.10, -0.11)** |  | -0.02  (-0.25, 0.21) | -0.07  (-0.26, 0.12) | -0.13  (-0.37, 0.10) | -0.08  (-0.27, 0.12) |  | -0.16  (-0.33, 0.01) | -0.16  (-0.35, 0.03) |  | -0.08  (-0.27, 0.11) | -0.13  (-0.33, 0.08) |  | **-0.19**  **(-0.37, -0.01)** |

*Note*: Generalized linear mixed models were used to estimate $\beta$ coefficients for the cognitive variables (MoCA-T scores or z scores of the cognitive domains). Adjusted for age, sex, education years, *APOE* ε4 status, depressive symptoms, follow-up time, practice effect, cigarette smoking, alcohol consumption, hypertension, diabetes mellitus, dyslipidemia, heart disease, physical activity by IPAQ, and interleukin-6 and C-reactive protein levels. The numbers in bold indicate significant findings (*p* < 0.05).

Abbreviations: MoCA-T, Montreal Cognitive Assessment-Taiwanese version; IPAQ, International Physical Activity Questionnaire.

**SUPPLEMENTARY TABLE 8**	Association between dynapenic obesity status and cognitive function, with obesity defined by fat mass index

| **Cognitive**  **function**  **Dynapenic**  **obesity**  **status** |  | **Global cognitive function** |  | **Logical memory** | | | |  | **Trail Making Test** | |  | **Digit span** | |  | **Verbal fluency** |
| --- | --- | --- | --- | --- | --- | --- | --- | --- | --- | --- | --- | --- | --- | --- | --- |
|  |  | MoCA-T  $\hat{\beta}$ (95% CI) |  | Immediate  theme recall  $\hat{\beta}$ (95% CI) | Immediate  free recall  $\hat{\beta}$ (95% CI) | Delayed  theme recall  $\hat{\beta}$ (95% CI) | Delayed  free recall  $\hat{\beta}$ (95% CI) |  | A  $\hat{\beta}$ (95% CI) | B  $\hat{\beta}$ (95% CI) |  | Forward  $\hat{\beta}$ (95% CI) | Backward  $\hat{\beta}$ (95% CI) |  | $\hat{\beta}$ (95% CI) |
| Nondynapenic-nonobese |  | Reference |  | Reference | Reference | Reference | Reference |  | Reference | Reference |  | Reference | Reference |  | Reference |
| Dynapenic-nonobese |  | **-0.46**  **(-0.87, -0.05)** |  | -0.13  (-0.32, 0.06) | -0.12  (-0.27, 0.04) | **-0.25**  **(-0.45, -0.06)** | **-0.22**  **(-0.38, -0.06)** |  | -0.09  (-0.22, 0.04) | **-0.21**  **(-0.37, -0.06)** |  | -0.12  (-0.28, 0.04) | -0.05  (-0.22, 0.12) |  | -0.11  (-0.26, 0.04) |
| Nondynapenic-obese |  | 0.07  (-0.43, 0.57) |  | -0.14  (-0.38, 0.10) | -0.15  (-0.37, 0.07) | **-0.25**  **(-0.50, -0.001)** | -0.16  (-0.38, 0.07) |  | -0.11  (-0.28, 0.06) | -0.16  (-0.37, 0.06) |  | -0.02  (-0.23, 0.20) | 0.01  (-0.21, 0.23) |  | -0.003  (-0.22, 0.21) |
| Dynapenic-obese |  | **-0.74**  **(-1.36, -0.12)** |  | -0.09  (-0.38, 0.21) | **-0.29**  **(-0.54, -0.03)** | -0.10  (-0.40, 0.20) | -0.17  (-0.43, 0.08) |  | -0.16  (-0.36, 0.05) | 0.04  (-0.21, 0.29) |  | -0.18  (-0.43, 0.07) | -0.07  (-0.33, 0.19) |  | -0.08  (-0.33, 0.16) |

*Note*: Generalized linear mixed models were used to estimate $\beta$ coefficients for the cognitive variables (MoCA-T scores or z scores of the cognitive domains). Adjusted for age, sex, education years, *APOE* ε4 status, depressive symptoms, follow-up time, practice effect, cigarette smoking, alcohol consumption, hypertension, diabetes mellitus, dyslipidemia, heart disease, physical activity by IPAQ, and interleukin-6 and C-reactive protein levels. The numbers in bold indicate significant findings (*p* < 0.05).

Abbreviations: MoCA-T, Montreal Cognitive Assessment-Taiwanese version; IPAQ, International Physical Activity Questionnaire.

**SUPPLEMENTARY TABLE 9**	Association between dynapenic obesity status and cognitive function, without exclusion of underweight participants (*n* = 373)

| **Cognitive**  **function**  **Dynapenic**  **obesity**  **status** |  | **Global cognitive function** |  | **Logical memory** | | | |  | **Trail Making Test** | |  | **Digit span** | |  | **Verbal fluency** |
| --- | --- | --- | --- | --- | --- | --- | --- | --- | --- | --- | --- | --- | --- | --- | --- |
|  |  | MoCA-T  $\hat{\beta}$ (95% CI) |  | Immediate  theme recall  $\hat{\beta}$ (95% CI) | Immediate  free recall  $\hat{\beta}$ (95% CI) | Delayed  theme recall  $\hat{\beta}$ (95% CI) | Delayed  free recall  $\hat{\beta}$ (95% CI) |  | A  $\hat{\beta}$ (95% CI) | B  $\hat{\beta}$ (95% CI) |  | Forward  $\hat{\beta}$ (95% CI) | Backward  $\hat{\beta}$ (95% CI) |  | $\hat{\beta}$ (95% CI) |
| Nondynapenic-nonobese |  | Reference |  | Reference | Reference | Reference | Reference |  | Reference | Reference |  | Reference | Reference |  | Reference |
| Dynapenic-nonobese |  | **-0.47**  **(-0.84, -0.10)** |  | -0.07  (-0.25, 0.10) | -0.09  (-0.23, 0.05) | -0.17  (-0.34, 0.01) | **-0.16**  **(-0.30, -0.02)** |  | -0.07  (-0.19, 0.06) | **-0.17**  **(-0.31, -0.04)** |  | **-0.15**  **(-0.29, -0.01)** | -0.07  (-0.22, 0.08) |  | -0.13  (-0.26, 0.01) |
| Nondynapenic-obese |  | -0.27  (-0.92, 0.38) |  | -0.14  (-0.45, 0.17) | -0.16  (-0.43, 0.12) | -0.25  (-0.57, 0.07) | -0.21  (-0.49, 0.07) |  | -0.19  (-0.44, 0.05) | **-0.34**  **(-0.61, -0.07)** |  | -0.02  (-0.29, 0.25) | -0.02  (-0.30, 0.25) |  | -0.10  (-0.37, 0.17) |
| Dynapenic-obese |  | **-1.15**  **(-1.99, -0.31)** |  | -0.27  (-0.67, 0.13) | **-0.42**  **(-0.76, -0.08)** | -0.16  (-0.56, 0.25) | -0.30  (-0.65, 0.05) |  | **-0.45**  **(-0.75, -0.14)** | -0.01  (-0.34, 0.33) |  | 0.08  (-0.26, 0.42) | -0.17  (-0.53, 0.18) |  | -0.08  (-0.42, 0.25) |

*Note*: Generalized linear mixed models were used to estimate $\beta$ coefficients for the cognitive variables (MoCA-T scores or z scores of the cognitive domains). Adjusted for age, sex, education years, *APOE* ε4 status, depressive symptoms, follow-up time, practice effect, cigarette smoking, alcohol consumption, hypertension, diabetes mellitus, dyslipidemia, heart disease, physical activity by IPAQ, and interleukin-6 and C-reactive protein levels. The numbers in bold indicate significant findings (*p* < 0.05).

Abbreviations: MoCA-T, Montreal Cognitive Assessment-Taiwanese version; IPAQ, International Physical Activity Questionnaire.
